# Development and validation of a pharmacogenomics reporting workflow based on the illumina global screening array chip

**DOI:** 10.3389/fphar.2024.1349203

**Published:** 2024-03-11

**Authors:** Pamela Gan, Muhammad Irfan Bin Hajis, Mazaya Yumna, Jessline Haruman, Husnul Khotimah Matoha, Dian Tri Wahyudi, Santha Silalahi, Dwi Rizky Oktariani, Fitria Dela, Tazkia Annisa, Tessalonika Damaris Ayu Pitaloka, Priscilla Klaresza Adhiwijaya, Rizqi Yanuar Pauzi, Robby Hertanto, Meutia Ayuputeri Kumaheri, Levana Sani, Astrid Irwanto, Ariel Pradipta, Kamonlawan Chomchopbun, Mar Gonzalez-Porta

**Affiliations:** ^1^ Nalagenetics Pte Ltd., Singapore, Singapore; ^2^ PT Genomik Solidaritas Indonesia, Jakarta, Indonesia; ^3^ Department Biochemistry and Molecular Biology, Faculty of Medicine, Universitas Indonesia, Jakarta, Indonesia

**Keywords:** SNP microarray, copy number variation (CNV) calling, microarray-based genotyping, pharmacogenomics, single nucleotide variant (SNV) calling

## Abstract

**Background:** Microarrays are a well-established and widely adopted technology capable of interrogating hundreds of thousands of loci across the human genome. Combined with imputation to cover common variants not included in the chip design, they offer a cost-effective solution for large-scale genetic studies. Beyond research applications, this technology can be applied for testing pharmacogenomics, nutrigenetics, and complex disease risk prediction. However, establishing clinical reporting workflows requires a thorough evaluation of the assay’s performance, which is achieved through validation studies. In this study, we performed pre-clinical validation of a genetic testing workflow based on the Illumina Global Screening Array for 25 pharmacogenomic-related genes.

**Methods:** To evaluate the accuracy of our workflow, we conducted multiple pre-clinical validation studies. Here, we present the results of accuracy and precision assessments, involving a total of 73 cell lines. These assessments encompass reference materials from the Genome-In-A-Bottle (GIAB), the Genetic Testing Reference Material Coordination Program (GeT-RM) projects, as well as additional samples from the 1000 Genomes project (1KGP). We conducted an accuracy assessment of genotype calls for target loci in each indication against established truth sets.

**Results:** In our per-sample analysis, we observed a mean analytical sensitivity of 99.39% and specificity 99.98%. We further assessed the accuracy of star-allele calls by relying on established diplotypes in the GeT-RM catalogue or calls made based on 1KGP genotyping. On average, we detected a diplotype concordance rate of 96.47% across 14 pharmacogenomic-related genes with star allele-calls. Lastly, we evaluated the reproducibility of our findings across replicates and observed 99.48% diplotype and 100% phenotype inter-run concordance.

**Conclusion:** Our comprehensive validation study demonstrates the robustness and reliability of the developed workflow, supporting its readiness for further development for applied testing.

## 1 Introduction

Pharmacogenomics (PGx) is a specialized field of medicine that explores the interplay between an individual’s genetic makeup and their response to medications (Pirmohamed, 2023). It investigates how genetic variants influence drug metabolism, efficacy, and safety, aiding in understanding and predicting individual responses to specific drugs. One prominent example of its application is found in the study of the cytochrome P450 family 2 (CYP2), an extensively researched and well-understood enzyme family responsible for metabolizing approximately 25% of available drugs (Goh et al., 2017). For example, individuals with loss-of-function alleles in *CYP2C19* exhibit reduced activation of the prodrug clopidogrel, while those with extra copies of *CYP2D6* genes may experience adverse effects from standard doses of codeine (Scott et al., 2013; Crews et al., 2014). Additional drug-metabolizing enzymes, such as DPYD, TPMT, NUDT15, and VKORC, as well as transporters like SLCO1B1, also constitute common PGx targets (Pirmohamed, 2023). By identifying and interpreting these genetic variations, PGx facilitates the development of personalized therapeutic strategies aimed at enhancing drug efficacy and minimizing adverse drug reactions (ADRs).

Studies have shown that up to 70% of ADRs have strong genetic associations (Chan et al., 2016; Swen et al., 2023), and the financial burden of trial-and-error prescriptions is estimated to be immense, amounting to USD 30 billion (Sultana et al., 2013). To date, consortia such as the Clinical Pharmacogenetics Implementation Consortium (CPIC) and the Dutch Pharmacogenetics Working Group (DPWG) have published genotype-based guidelines for over a hundred gene-drug pairs, providing a robust and evidence-backed framework to facilitate the integration of PGx into everyday clinical practice (Relling and Klein, 2011; Bank et al., 2018; Relling et al., 2020; Abdullah-Koolmees et al., 2021). Remarkably, it is estimated that over 90% of the population carries at least one actionable pharmacogenomic variant, indicating the vast potential of PGx testing in guiding drug therapy and reducing the risk of ADRs (Dunnenberger et al., 2015; Pirmohamed, 2023). Thus, when contemplating a broad implementation of PGx testing, it is important to select a technology that is both widely accessible and cost-effective. Additionally, as outcomes of the tests can be used to guide therapeutic decisions, it is important to establish the analytical and clinical validity of the results before implementing them into patient care. For many molecular tests, assessing the analytical performance is relatively simple, as they yield binary outcomes like positive or negative results. However, PGx markers present a spectrum of genotypes which, when combined, can lead to diverse phenotypes. For example, CYP enzyme metabolizer phenotypes can range from “poor metabolizers” to “ultra-rapid metabolizers”. Therefore, careful consideration needs to be taken when designing validation studies to endorse the use of PGx tests (Huebner et al., 2023).

In this study, we describe the development and validation of a clinical reporting workflow for pharmacogenomics testing. This workflow uses the Illumina GSA chip, a widely available and cost-effective genetic testing solution, to report on 503 distinct variants across 25 PGx genes associated with 303 clinically actionable drugs. While previous studies have described the use of the GSA chip for PGx reporting (e.g., Reisberg et al., 2019), they did not include an accuracy assessment based on well-established reference materials. Our study addresses this gap by providing a comprehensive analytical validation of the Illumina GSA chip, specifically focusing on its application in preemptive pharmacogenomics testing.

## 2 Materials and methods

### 2.1 PGx reporting workflow and validation study design

**FIGURE 1:**
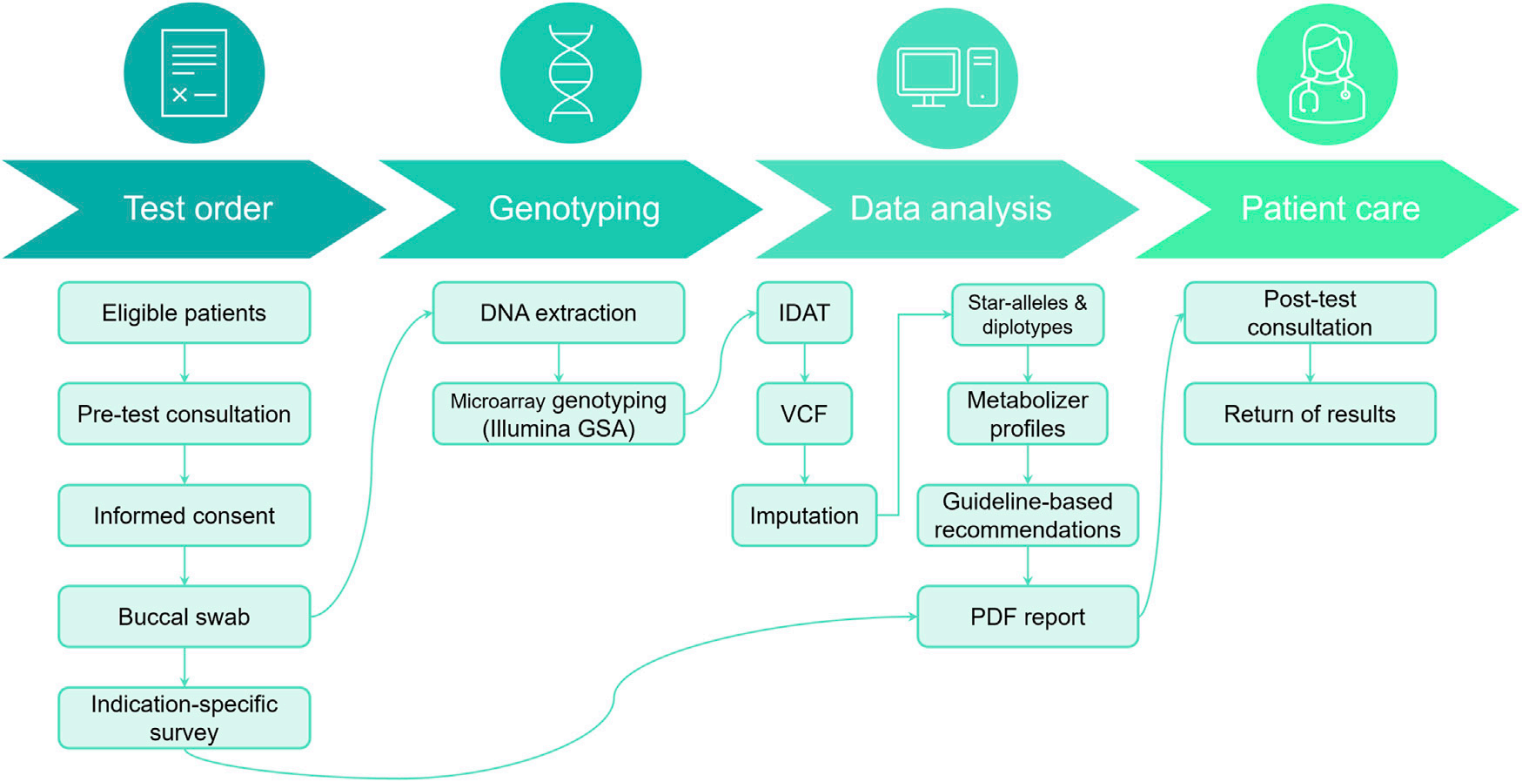
Patient journey for PGx testing workflow. During the pre-test consultation, patients provide informed consent, complete a PGx survey, and submit a DNA sample from a buccal swab. This sample undergoes DNA extraction and array genotyping, followed by bioinformatic analysis to characterize variants in selected PGx genes. Genotype calls are subsequently interpreted into metabolizer profiles and annotated with actionable recommendations from published guidelines. Finally, results are compiled into a PDF report, which is discussed with the patient during a post-test consultation.

We have developed a pharmacogenomics reporting workflow centered around the Illumina GSA v3 chip (see Figure 1 and Methods). The patient journey begins with a pre-test counseling session, during which eligibility for the pharmacogenomics (PGx) test is determined by a qualified physician. Typically, this test is recommended for patients who are currently taking the medications being examined, those about to start treatment, or those with a specific interest. During this session, eligible participants receive an overview of the test’s purpose and procedure.

Following this informative session, participants provide consent and submit buccal samples that will be used during the genotyping process. In addition, they also complete an indication-specific survey detailing their current medications, information that will be used during the post-test consultation discussion. The buccal swab sample is then sent to a clinically accredited laboratory for DNA extraction and genotyping (see Section 2.2). Finally, a PDF report is generated and provided to the patient during a post-test consultation (example recommendation: Supplementary File S1).

**FIGURE 2:**
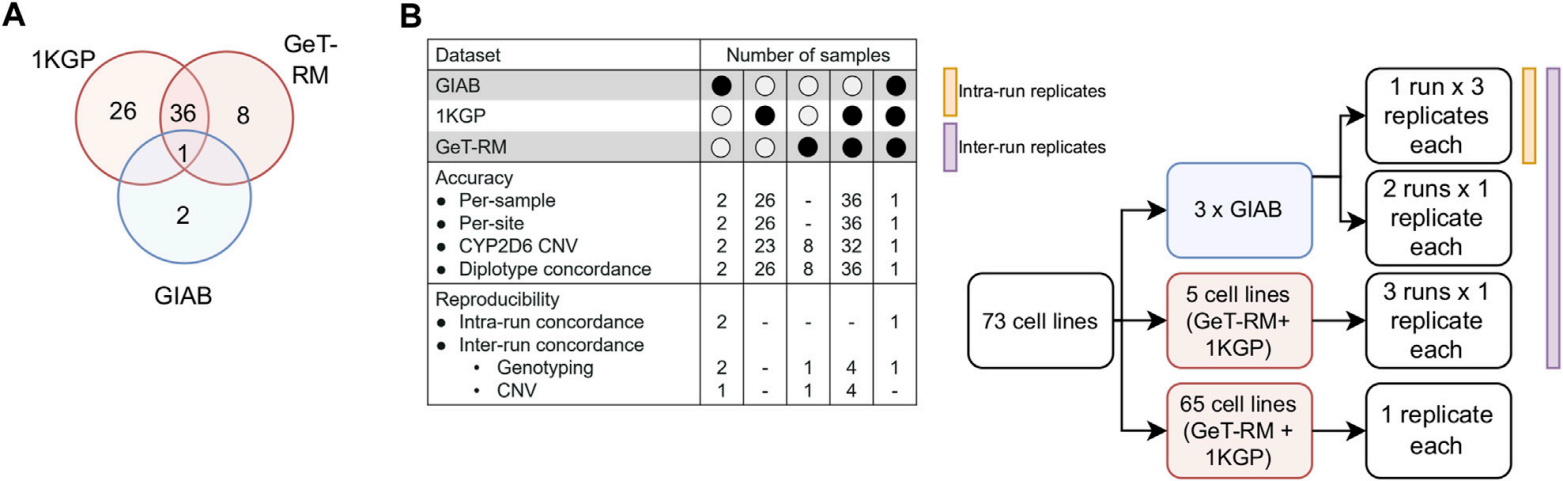
Validation study design. **(A)**. DNA from a total of 73 unique reference cell lines were genotyped on the GSA chip to assess genotyping accuracy. Samples were selected due to the availability of well-characterized reference genotype calls (1KGP, GIAB) or reference calls for important PGx genes that have been validated experimentally by multiple labs (GeT-RM). **(B)**. Breakdown of samples by experiment. GIAB samples were ran in triplicate in a 3:1:1 design to enable measurement of inter- and intra-run reproducibility. Selected GeT-RM samples with known copy number variations (CNV) were also run across three runs to assess inter-run reproducibility of CNV calling.

Our reporting workflow encompasses a total of 25 pharmacogenes and 503 distinct variants corresponding to 429 haplotypes (Supplementary Tables S1, S2). These genes have been selected to include the Very Important Pharmacogenes listed in PharmGKB which overlap with markers in the Illumina GSA chip (N = 21 out of 35 Tier 1 VIPs) (Whirl-Carrillo et al., 2012). To validate the accuracy of the test results, we designed a comprehensive validation study using well-established reference materials, selected to represent a broad range of PGx outcomes (Figure 2; Supplementary Table S3). Specifically, we utilized three GIAB cell lines, 45 cell-lines from the United States Centers for Disease Control and Prevention (CDC) Genetic Testing Reference Material (GeT-RM) Coordination Program (Pratt et al., 2010), including 37 samples that are also part of the 1000 Genomes Project (1KGP) (Byrska-Bishop et al., 2022), and an additional 26 cell lines from the 1KGP to conduct six distinct experiments, which included assessments of accuracy (per-sample, per-site, *CYP2D6* copy number variation (CNV) calling and star allele concordance) as well as intra- and inter-run reproducibility. Importantly, among the 429 haplotypes included in the reportable range of our test, 84 could be directly tested under this experimental design.

### 2.2 Samples and genotyping

The three Genome-In-A-Bottle (GIAB) DNA samples were obtained from the National Institute of Standards and Technology (NIST). In addition, a total of 70 DNA samples were purchased from the Coriell Institute for Medical Research, (https://www.coriell.org). These samples were selected to cover an array of ethnicities and pharmacogenes (Supplementary Table S2).

Infinium Global Screening Array-24 v3.0 BeadChips were processed according to the standard Infinium High-throughput Screening (HTS) protocol according to manufacturer’s instructions. Signal intensities were converted into idat files using an iScan^®^ machine (Illumina Inc.).

For CNV calling, B-allele frequency (BAF) and log normalized intensity ratio (LRR) data were generated using GenomeStudio v2.0.5 (Illumina Inc.) with a custom cluster file created according to the manufacturer’s instructions. Array-based sample genders were also estimated in this step.

### 2.3 Data processing

#### 2.3.1 Quality control

For genotyping, idat files were converted to gtc format based on human genome build GRCh38. p13 using the Illumina Array Analysis Platform Genotyping Command Line Interface (iaap cli) (v1.1.0) followed by conversion to VCF using the python script gtc_to_vcf.py (v1.2.1) (https://github.com/Illumina/GTCtoVCF).

Chip-wide autosomal call rates were calculated using plink2 (v alpha3.7) and only samples with greater than 0.98 call rate and whose array-based genders matched to the expected genders were processed. Sites with >90% missing calls and with less than 0.5% minor allele frequency were removed prior to phasing with Eagle2 (Loh et al., 2016) and imputation with minimac4 (Das et al., 2016) using the 1KGP Phase 3 (Byrska-Bishop et al., 2022) data as a reference panel. The final set of genotypes used for diplotype calls includes both directly genotyped as well as imputed calls with genotype likelihoods above 0.8. Where a site has both a genotyped and imputed call, the directly genotyped site is utilized for diplotype calling instead of the imputed call.

#### 2.3.2 CNV calling

CNV calls were made with PennCNV (Wang et al., 2007) using a custom PFB (Population frequency of B allele) file and GC-content model generated using scripts from PennCNV. CNV calls for samples with LRR standard deviation greater than 0.2, BAF drift greater than 0.01 and a wave factor greater than 0.05 after GC model adjustment are not considered further. CNV calls supported by less than 10 probes or covering less than 250 bp were removed using the filter_cnv.pl script from PennCNV.

Reference CNV calls were obtained based on GeT-RM and PacBio calls (Chen et al., 2021). In addition, reference calls for WT copy number, whole gene duplications and deletions were obtained from a 1KGP WGS analyzed dataset generated in a previous study from Lee et al. (2022) (Lee et al., 2022). When a duplication is detected, the *CYP2D6* allele that is duplicated cannot be assigned based on the available information and is reported as “copy number≥3” in order to indicate that while a duplication is detected, the exact duplicated allele cannot be specified.

#### 2.3.3 Diplotype calling

Diplotype calls for *CYP2D6, CYP2C19, CYP2B6, CYP2C9, CYP3A5*, *CYP4F2* and *SLCO1B1* were made using a combination of PharmCAT (v2.2.3) (Sangkuhl et al., 2020) and in-house custom scripts to resolve ambiguous genotypes. When more than one diplotype is possible based on the genotyped SNPs, this is classified as an “ambiguous” call and the call is resolved where possible based on the frequency in the population to which the sample belongs. In addition, the metabolizer profile is classified as a “Possible” call to indicate that other calls may be possible based on the information acquired. For *CYP2D6*, in-house scripts were also used to make additional adjustments based on copy number changes detected: *5 reported when a deletion was detected, or “copy number≥3” added when duplications were detected. Calls for other genes were generated using in-house scripts based on allele tables from PharmGKB.

Additionally, pypgx ver 0.20.0 (Lee et al., 2022) was utilized by running the “run-chip-pipeline” with the parameter: --assembly GRCh38.

#### 2.3.4 Metabolizer profiles

In general, for genes with diplotype calls, metabolizer profiles were assigned based on diplotype calls referring to a curated set of gene, diplotype and phenotype calls obtained from PharmGKB. For samples with *CYP2D6* duplications, metabolizer profiles were assigned after calculation of copy number (Crews et al., 2014) using in-house scripts. In such a case, the activity score for duplications of either haplotype is calculated assuming a maximum of three *CYP2D6* copies and a range of activity scores is obtained (i.e., activity score for two copies of star allele 1 and one copy of star allele 2 as well as one copy of star allele 1 and two copies of star allele 2). Based on the minimum and maximum values of the activity scores, the metabolizer phenotype is assigned. If the activity scores fall within the ranges of two different metabolizer profiles, the activity score of the more frequent combination of all possible copy number permutations of the two star alleles according to PharmGKB is reported. In all cases, the word “possible” is added to the metabolizer profile to indicate that another metabolizer profile is still possible.

### 2.4 Assessment of Accuracy and precision

Genotyping accuracy was assessed using GIAB samples (HG001, HG002, HG005) and 1KGP samples. For 1KGP samples, genotype calls from the 30× 1,000 Genome Phase 3 Reanalysis with DRAGEN 3.7.6 accessed from https://registry.opendata.aws/ilmn-dragen-1kgp on 3 November 2023 were used as the truth set. For GIAB samples, DeepVariant genotype calls from UltimaGenomics accessed on 7 June 2023 (GIAB, 2023) were used as the truth set. Variant calls were classified as true positive, true negative, false positive and false negative as previously described (Kishikawa et al., 2019). Discordant calls in *CYP2D6*, which is known to have many structural variants, and *G6PD*, on the X chromosome, were adjusted manually, if supported by external information (expected copy numbers or gender).

The following metrics were calculated as such:

• Callability: The percentage of successfully genotyped loci out of all considered genotypes.
• Genotype concordance: The percentage of genotyped sites with a correct call.
• Analytical sensitivity: The percentage of variant sites correctly identified.
• Analytical specificity: The percentage of non-variant sites correctly identified.
• Precision: The percentage of variants correctly genotyped relative to the number of reported variants.
• No-call rate: Percentage of missing genotypes out of all considered genotypes.

Per-site concordances were calculated using the same definitions for all 503 variants on a per-site basis out of 65 samples per site.

For diplotype concordance, concordance was defined as the percentage of samples with a correct call out of samples with a reference call. For *UGT1A1*, *CYP2D6* and *SLCO1B1* genes, diplotype calls that differed between the reference dataset calls and our pipeline were still considered concordant if appropriate based on the pipeline’s reportable range. Specifically, *1 and *2 for *CYP2D6* were evaluated as equivalent as the key variant for *2 is not directly genotyped, in the absence of an imputed call, will default to *1. For *UGT1A1*, *60 was considered equivalent to *1. Further, as only *80 was genotyped, a call for *80 was considered to be concordant with calls for *28 and *37 in the reference set. As in the PharmGKB *UGT1A1* notes (version: 04/28/2023), as only *80 is tested, in the report, the decreased function of *80 based on its high linkage equilibrium with *28 and *37 is inferred, although it is noted that it is not 100%. For *CYP2C19*, *38, which is the reference allele reported in the absence of any mutation, was considered equivalent to *1. The truth set for *CYP2B6, CYP2C19, CYP2C8, CYP2C9, CYP2D6, CYP3A4, CYP3A5, CYP4F2, TPMT*, and *UGT1A1* diplotype calls were obtained from GeT-RM. However, for *DPYD, G6PD, NUDT15*, and *SLCO1B1*, the truth sets were PharmCAT calls based on 1KGP NGS VCFs. This was due to either a scarcity of samples with GeT-RM calls in the validation set or updates in the haplotype definitions since the GeT-RM studies were conducted (*SLCO1B1* and *DPYD*). Diplotypes were considered concordant as long as there were samples with consistent calls in the truth sets.

Confidence intervals for point estimates of the above metrics were estimated using the Wilson score interval (Wilson, 1927; Newcombe and Altman, 2011). Confidence intervals for means of the above metrics were estimated by bootstrapping (n = 10,000).

#### 2.4.1 Allele frequencies

Alleles frequencies were obtained from the Phase 31,000 Genomes dataset described above. Samples were subsetted by population and allele frequencies were calculated using bcftools (v1.9) +fill-tags. In addition, population level aggregated allele frequencies per-site were obtained from the Tohoku Medical Megabank project (Kuriyama et al., 2016; Tadaka et al., 2023).

## 3 Results

### 3.1 Accuracy assessment of genotype calls

First, we aimed to assess the accuracy of our genotyping and imputation workflow by evaluating our ability to obtain correct genotype calls at individual PGx loci. We conducted two complementary analyses: one centered on per-site assessments and the other on per-sample evaluations.

In the per-site analysis, we evaluated variant calling performance for 503 PGx loci, including 33 imputed sites (Supplementary Table S1) in the 65 samples of the validation set with reference genotype calls. Out of 503 sites, 278 loci had a call in the 1KGP Phase 3 reference set. Of these, 114 sites (41.00%) could be evaluated with a true positive with the genotyping validation set. For the remaining sites, only two loci (rs114096998 in *DPYD* and rs34223104 in *CYP2B6*) are present in the 1KGP dataset with an allele frequency of higher than 1% and 15 loci (rs35350960 in *UGTA1* genes; rs55951658 in *CYP3A4*; rs2306282, rs72559747 and rs71581941 in *SLCO1B1*, rs72559747 rs186364861 in *NUDT15*; rs1135835, rs1135833, rs72549352, rs567606867, rs567606867 and rs118203758 in *CYP2D6*; and rs72554664, rs72554665, rs137852342 and rs137852327 in *G6PD*) have an allele frequency of higher than 1% in at least one of the East Asian populations represented in the dataset (CHS, CDX, KHV, CHB and JPT). Further, only 18 of the unassessed SNPs had a minor allele frequency of greater than 0.1% in the Tommo 54K dataset consisting of 54,300 Japanese individuals, indicating that the majority of unevaluated sites are present at low frequencies in Asian populations.

**FIGURE 3:**
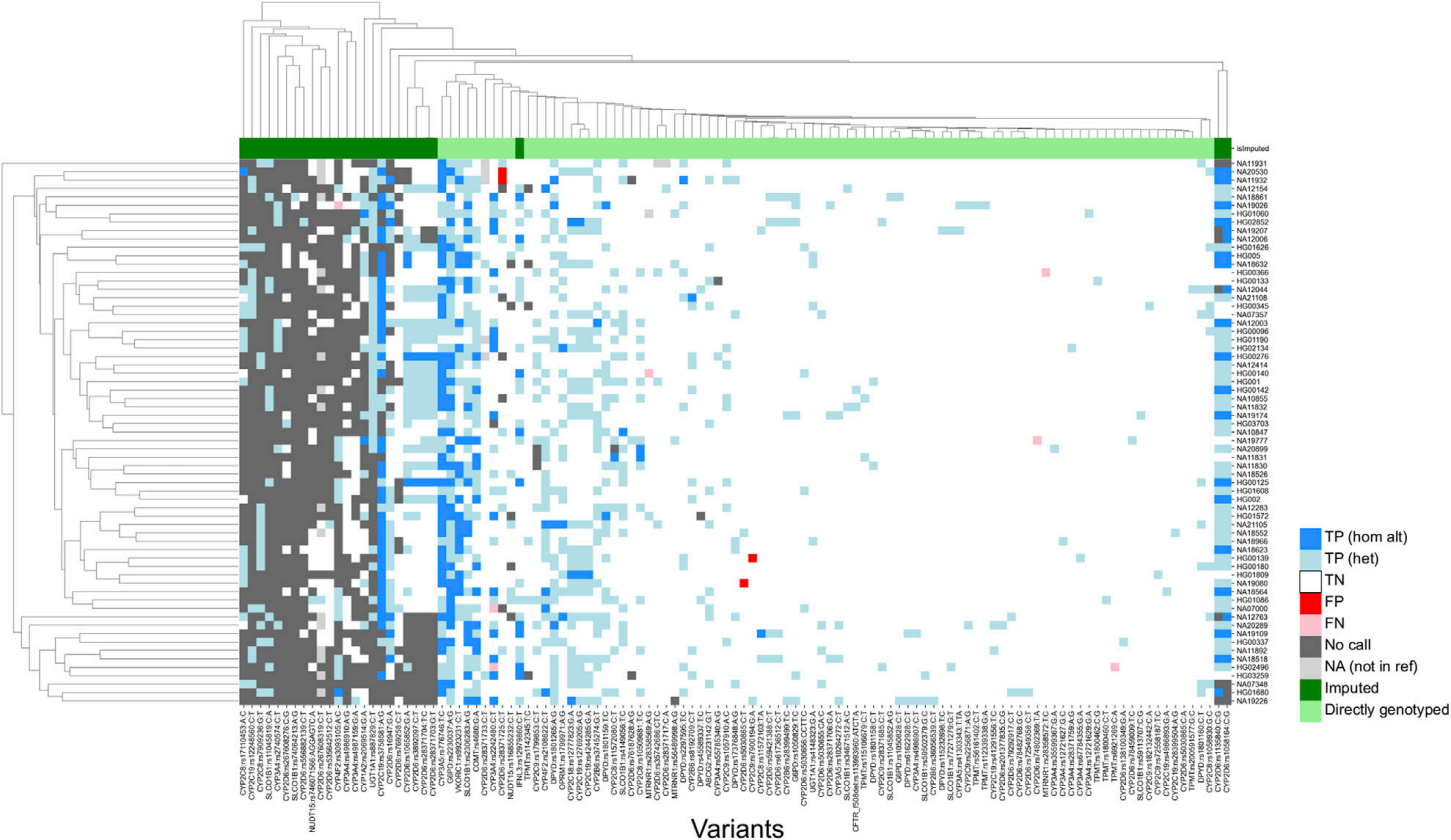
Genotyping concordance against 65 accuracy controls (per-site analysis). Heatmap showing concordance of 114 variants with true positives. 1KGP and GIAB samples were utilized as accuracy controls. Imputed sites have a lower callability compared to sites that are directly genotyped. TP (hom alt), True positive homozygous alternate; TN (het), true positive heterozygous; TN, True negative; FP, false positive; FN, false negative; NA, not in reference.

Of the 114 sites that could be evaluated, only four (rs1135840, rs1058164 and rs1065852 in *CYP2D6*; and rs3093105 in *CYP4F2*) had a concordance of less than 95%. Among these, for the three loci associated with *CYP2D6*, discrepancies could be attributed to differences in query versus reference dosages. For these sites, the discordant samples were known to harbor structural variants of *CYP2D6*, and the dosages reported in the reference calls were not consistent with the expected diplotypes. For example, all three were reported to be homozygous alternate (dosage = 2) in the 1KGP dataset for HG01190 and NA18861, which are known to have only a single copy of the *CYP2D6* gene each, while the microarray results reported the expected dosage of 1. This led to the calls being labelled as false negatives, due to the discordance with the truth data. Further, rs1058164 for NA19207, which has a duplicated copy of *CYP2D6* *2, was reported as heterozygous according to the reference VCF, whereas the microarray results reported the expected dosage of 2 for the same SNP, thus resulting in misclassification as a false positive. After adjustment, the results indicated that 99.80% of sites (502/503) exhibited 95% concordance with the expected calls (Figure 3), thus demonstrating a high level of per-site accuracy in our workflow.

After manual review of discordant calls, our results revealed consistently high analytical sensitivity and specificity across all tested samples, with means of 99.39% [95%CI = 91.67–100.00] and 99.98 [95%CI = 99.79–100.00], respectively (Table 1). Altogether, these findings demonstrate the applicability of our workflow in accurately determining genotypes for small variants at PGx loci.

**TABLE 1:** Per-sample accuracy assessment (SNPs and INDELs). Mean sample callability, genotype concordance, analytical sensitivity, analytical specificity, precision and no-call rates of 65 samples, focusing on 503 loci, with 95% confidence intervals estimated by bootstrapping.

Metric	Mean (%)
Callability	96.08 [94.11, 97.45]
Genotype concordance	99.96 [99.79, 100.00]
Analytical sensitivity	99.39 [91.67, 100.00]
Analytical specificity	99.98 [99.79, 100.00]
Precision	99.54 [93.75, 100.00]
No-call rate	3.92 [2.55, 5.89]

Additionally, we conducted an extensive evaluation of the *CYP2D6* gene, which encodes the Cytochrome P450 2D6 enzyme responsible for metabolizing approximately 25% of clinically used drugs. This gene exhibits significant genetic diversity among individuals, including structural variants and complex events like hybrid rearrangements (Zhou et al., 2017; Gaedigk et al., 2018). The presence of two pseudogenes in the human genome further complicates genotyping efforts. In this study, our primary objective was to assess the capability of our assay to accurately genotype complex variants within the *CYP2D6* gene. To achieve this, we included 22 cell lines with challenging *CYP2D6* haplotypes into our experimental design, encompassing whole gene deletions, duplications, and complex events such as hybrid rearrangements and co-occurring deletions and duplications. Based on this dataset, we estimate the analytical sensitivity and specificity for CNVs in *CYP2D6* gene to be 60.00% [95% CI = 35.75–80.18] and 100.00% [95% CI = 89.85–100.00], respectively (Table 2). To troubleshoot the observed decrease in analytical sensitivity, we divided the performance assessment by structural variant type. We identified that the drop in performance was primarily driven by hybrid tandem duplications, where analytical sensitivity was 0.00% (95% CI = 0.00, 39.03), compared to 90.91% (95% CI = 62.26, 98.38) for duplications and deletions (Table 2).

**TABLE 2:** CYP2D6 structural variation detection accuracy assessment**.** Concordance, sensitivity, specificity and precision metrics are shown along with the 95% confidence intervals. The assay had a callability of 78.79% [95%CI = 67.49–86.92] and a no-call rate of 21.21% [95%CI = 13.08–32.51] across the 66 cell lines included in the analysis.

Structural variation class	Number in reference set	Genotype concordance (%)	Analytical sensitivity (%)	Analytical specificity (%)	Precision (%)
All	22	88.46 [77.03, 94.60]	60.00 [35.75, 80.18]	100.00 [90.59, 100.00]	100.00 [70.09, 100.00]
Duplication + Deletion	17	98.08 [89.88, 99.66]	90.91 [62.26, 98.38]	100.00 [91.43, 100.00]	100.00 [72.25, 100.00]
Duplication (xN)	9	98.08 [89.88, 99.66]	85.71 [48.69, 97.43]	100.00 [92.13, 100.00]	100.00 [60.97, 100.00]
Deletion (*5)	8	100.00 [93.12, 100.00]	100.00 [51.01, 100.00]	100.00 [92.59, 100.00]	100.00 [51.01, 100.00]
Hybrid tandem duplication	8	88.46 [77.03, 94.60]	0.00 [0.00, 39.03]	100.00 [92.29, 100.00]	N/A

### 3.2 Concordance of PGx star alleles

Next, we proceeded to evaluate diplotype calling accuracy in 14 out of 25 pharmacogenes that are reported as star-alleles in our test (Supplementary Table S2). An inherent challenge of this analysis lies in the extensive number of star-alleles associated with each pharmacogene, best exemplified by *CYP2D6*, which includes over 100 known star-alleles in PharmGKB (Zhou et al., 2017; Gaedigk et al., 2018). As a result, finding reference materials to cover each of the alleles during validation is a challenging task, and some alleles (e.g., population-specific ones or novel additions) may not even be present in the current reference material resources. To address this challenge, we selected samples from the GeT-RM database to cover as many samples as possible with the highest diversity of star alleles for the pharmacogenes in our test, prioritizing frequent haplotypes. We curated a final validation set of 73 samples, covering star-alleles for 84 out of 429 possible haplotypes that can be identified by the 503 sites in our test, and achieving a coverage rate of 35.40%. Among the genes considered, *UGT1A1* displayed the highest coverage level, while *G6PD* had the lowest coverage (3.39%), aligning with known variation and lack of reference data in GeT-RM, where only two out of the 186 non-reference haplotypes are represented, and where the majority of “haplotypes” are single SNP calls (McDonagh et al., 2012).

Having identified an appropriate sample set that maximizes star-allele representation, we proceeded to genotype each sample using our PGx workflow and subsequently evaluated the concordance of predicted diplotype calls against their respective truth sets. Two sources of truth sets were utilized: GeT-RM calls were used as the truth set for *CYP2B6, CYP2C19, CYP2C8, CYP2C9, CYP2D6, CYP3A4, CYP3A5, CYP4F2, TPMT* and *UGT1A1*. For *DPYD*, *G6PD*, *NUDT15* and *SLCO1B1*, truth sets were generated based on 1KGP NGS VCFs run on PharmCAT and pypgx. For *NUDT15* and *G6PD*, this was due to the limited number of samples with GeT-RM calls among the validation set, and for *DPYD* and *SLCO1B1*, the 1KGP dataset was used as a reference based on PharmCAT calls due to updates in the haplotype definitions since the GeT-RM studies were carried out. Per-gene concordances were calculated using only samples that had calls with either the GeT-RM or PharmCAT 1KGP reference truth sets (Table 3). For genes that were evaluated against PharmCAT calls (*NUDT15, G6PD, DPYD* and *SLCO1B1*), samples assessed with another diplotype caller, pypgx (Lee et al., 2022). The results for the comparison against pypgx were consistent: 93.44% concordance for calls from the current pipeline for *SLCO1B1* compared to both callers, and 100% concordance against *G6PD, NUDT15* and *DPYD* (Supplementary Table S4). For *DPYD*, calls were considered concordant if the same mutations were reported. For example, the call was considered concordant for HG01608, where pypgx identified haplotype 1 as Reference and haplotype 2 as c.1627A>G (*5); c.85T>C (*9A) and the current pipeline reported c.85T>C (*9A)/c.1627A>G (*5).

**TABLE 3:** Concordance of PGx star alleles**.** Diplotype and phenotype callability and concordance for 14 genes with haplotype calls. For *UGT1A1*, genotype concordance was adjusted by assuming *60 is equivalent to *1 and that *80 is equivalent to *27 and *37. For *CYP4F2*, the phenotype concordance is assessed based on if *3 is present or absent since *3 is used for the drug recommendation.

Gene	Size of reference call set	Diplotype callability (%)	Diplotype concordance (%)	Phenotype callability (%)	Phenotype concordance (%)
*CYP2B6*	27	88.89 [71.94, 96.15]	95.83 [79.76, 99.26]	88.89 [71.94, 96.15]	95.83 [79.76, 99.26]
*CYP2C19*	27	96.30 [81.72, 99.34]	92.31 [75.86, 97.86]	96.30 [81.72, 99.34]	92.31 [75.86, 97.86]
*CYP2C8*	27	100.00 [87.54, 100.00]	100.00 [87.54, 100.00]	NA [NA, NA]	NA [NA, NA]
*CYP2C9*	27	92.59 [76.63, 97.94]	100.00 [86.68, 100.00]	92.59 [76.63, 97.94]	100.00 [86.68, 100.00]
*CYP2D6*	40	80.00 [65.24, 89.50]	87.50 [71.93, 95.03]	90.00 [76.95, 96.04]	97.22 [85.83, 99.51]
*CYP3A4*	27	100.00 [87.54, 100.00]	100.00 [87.54, 100.00]	100.00 [87.54, 100.00]	100.00 [87.54, 100.00]
*CYP3A5*	27	100.00 [87.54, 100.00]	100.00 [87.54, 100.00]	100.00 [87.54, 100.00]	100.00 [87.54, 100.00]
*CYP4F2*	25	100.00 [86.68, 100.00]	92.00 [75.03, 97.78]	100.00 [86.68, 100.00]	100.00 [86.68, 100.00]
*DPYD*	43	86.05 [72.74, 93.44]	100.00 [90.59, 100.00]	90.70 [78.40, 96.32]	100.00 [91.03, 100.00]
*NUDT15*	64	100.00 [94.34, 100.00]	100.00 [94.34, 100.00]	100.00 [94.34, 100.00]	100.00 [94.34, 100.00]
*SLCO1B1*	63	96.83 [89.14, 99.13]	93.44 [84.32, 97.42]	98.41 [91.54, 99.72]	100.00 [94.17, 100.00]
*TPMT*	31	100.00 [88.97, 100.00]	93.55 [79.28, 98.21]	100.00 [88.97, 100.00]	93.55 [79.28, 98.21]
*UGT1A1*	25	100.00 [86.68, 100.00]	96.00 [80.46, 99.29]	100.00 [86.68, 100.00]	96.00 [80.46, 99.29]
*G6PD*	63	100.00 [94.25, 100.00]	100.00 [94.25, 100.00]	100.00 [94.25, 100.00]	100.00 [94.25, 100.00]

The results of this analysis revealed an average diplotype concordance of 96.47% across the 14 genes assessed (Table 3). Notably, certain genes, including *NUDT15, DPYD, G6PD, UGT1A1, CYP3A5, CYP3A4, CYP2C8,* and *CYP2C9*, displayed a high degree of concordance consistent with the relatively low number of variants under consideration (average of three diplotypes), in part due to other genotypes being relatively rare.

In contrast, the *CYP2D6* gene displayed the lowest diplotype concordance (87.50%), which was expected due to the aforementioned challenges. Specifically, fusion or hybrid haplotypes, such as *10+*36, *68+*4, were identified as *10 or *4, respectively, which aligns with the lowest performance in detecting CNVs previously discussed. In addition, rarer star alleles such as *15, *82, *17 and *56 were either not identified or were reported as wild type (the absence of a reportable mutation). Further, for *SLCO1B1*, *37/*37 was incorrectly called as *14/*37 in five samples due to the inability to resolve the correct diplotype based on the expected frequency in the population.

Importantly, when interpreting diplotypes into metabolizer profiles, phenotype concordance improved across all genes, with an average observed value of 98.07% (Table 3). The same improvement was also detected for *CYP2D6* (97.22% phenotype concordance), despite the diplotype call differences due to hybrid/tandem duplications described above. This is most likely due to the most common *CYP2D6* hybrid tandem duplications being associated with star alleles with similar allele functions. For example, *10+*36, which is the fusion of a no-function *36 allele and a decreased function *10 allele, is itself a decreased function allele, which is functionally the same as the *10 allele detectable by the pipeline. Another example is *68+*4 (no function allele), which is functionally the same as *4 only, that is reportable by the pipeline. For *SLCO1B1*, *14/*37 and *37/*37 both evaluated as normal function and overall, the phenotype concordance was 100.00%. The lowest phenotype concordance was *CYP2C19* (92.31%).

For *CYP2C19*, there were two samples with discordant diplotype calls out of the 26 samples assessed with truth set calls (92.31% concordant). The mismatched calls were for NA19109 (reported as *38/*38 Possibly Normal Metabolizer, while *17/*17 Ultrametabolizer is the expected call) and NA19178 (*38/*38 Possibly Normal Metabolizer reported while *1/*6 Intermediate Metabolizer is the expected call). The *17 haplotype is identified by a mutation in rs12248560. In this pipeline, this site is imputed and was a no call in the case of NA19109. For NA19178, *6 is defined by rs72552267, a site that is directly genotyped by the GSA chip. In the absence of calls in these two cases with two separate underlying causes, the *CYP2C19* WT star allele was reported (i.e., *38). In addition, the pipeline reported these as ‘Possibly Normal Metabolizer’s) to indicate the potential of another metabolizer profile being possible.

Apart from this, it was noted that *CYP2C9, CYP2B6* and *DPYD* have diplotype callabilities of less than 95% (92.59, 88.89% and 86.05% respectively) although the diplotype concordances are still high (100, 95, 100% respectively). In these cases, this was due to multiple potential diplotype calls which could not be resolved resulting in no calls.

### 3.3 Precision (reproducibility) study

Finally, we conducted a precision study to evaluate the reproducibility of our workflow, considering both intra-run and inter-run consistency at both the genotype and diplotype levels.

To assess intra-run precision of genotype calls, we utilized three GIAB samples (HG001, HG002, and HG005), each ran in triplicate, achieving 100% concordance across all replicates (Supplementary Table S5). In the inter-run evaluation of genotype calls, we expanded the dataset to include five additional GeT-RM samples, also ran in triplicate, consistently observing 100.00% concordance across all runs (Supplementary Table S5). These additional GeT-RM samples were deliberately selected because they harbor known duplications and deletions in CYP2D6. Further analysis of intra-run performance for *CYP2D6* diplotype calling confirmed 100.00% diplotype calling concordance across all runs (Supplementary Table S6). Lastly, by expanding the analysis to include all reported genes, we identified an average of 100.00% intra-run concordance and 99.50% inter-run concordance (Supplementary Table S6). The slight reduction in inter-run genotype concordance was influenced by differences in variants called for *MT-RNR1* between runs of NA19226. However, phenotype concordance was 100.00% between all samples, including for *MT-RNR1* of NA19226 where all samples were assigned a Normal Risk based on the results.

## 4 Discussion

Pharmacogenomics (PGx) is revolutionizing personalized medicine by providing insights into individual drug responses based on genetics. This approach has the potential to significantly improve treatment outcomes, reduce adverse drug reactions, and ultimately lower treatment costs (Pirmohamed, 2023). It is estimated that over 90% of the population carries at least one actionable pharmacogenomic variant (Dunnenberger et al., 2015; Pirmohamed, 2023). Furthermore, PGx information is already actionable: the DPWG and CPIC consortia have to date curated dosing guidelines based on genetics for over 140 drugs (Bank et al., 2018; Abdullah-Koolmees et al., 2021). As such, with the increasing incorporation of PGx information into patient care, there emerges a pressing need for the development and validation of routine PGx tests. Our study introduces a pre-emptive PGx reporting workflow that utilizes the widely available Illumina GSA chip, covering 503 variants across 25 pharmacogenes, and including 21 out of the 35 Tier 1 Very Important Pharmacogenes listed in PharmGKB (those with markers in the Illumina GSA chip).

To assess the accuracy and reliability of our test, we designed a comprehensive validation study, incorporating a selection of well-established reference materials from the GIAB (Springer Nature, 2015) and GeT-RM consortia (Pratt et al., 2010; 2016; 2022; Gaedigk et al., 2022). Additionally, we included cell lines from the 1000 Genomes Project (Byrska-Bishop et al., 2022) to address loci not covered by the aforementioned resources, but that were part of the reportable range of our test. In total, our study comprised 73 unique samples, strategically chosen to maximize the representation of star-alleles across the 25 genes covered by our test. It complements previous studies utilizing the GSA chip for PGx reporting (Reisberg et al., 2019) by providing a comprehensive performance evaluation based on well-established reference materials.

We analyzed the results from this sample set to establish the analytical performance of the assay, including per-sample and per-site accuracy, CNV calling performance in *CYP2D6*, star allele concordance, and as intra- and inter-run reproducibility. From these studies, and focusing on the target 503 PGx loci, we determined the assay’s mean analytical sensitivity to be 99.39% [95%CI = 91.67–100.00] and the analytical specificity to be 99.98% [95%CI = 99.79–100.00]. To complement these performance metrics, and identify systematic sources of error at each locus, we also conducted a more detailed per-site analysis, evaluating each target site across all samples in the validation set. We observed that 99.80% of loci (502/503) consistently demonstrated concordance with the expected calls across samples. Next, following the interpretation of genotype calls into star-alleles and diplotype calls, we continued our assessment by comparing diplotype results to those in the truth set. On average, for the 14 genes with haplotypes, the diplotype concordance was 96.47%. Notably, the evaluation of a subset of samples in replicate settings yielded consistent results, with an average 99.48% inter-run and 100% intra-run concordance rates. Overall, our workflow was demonstrated to exhibit both accuracy and precision. When compared to other array-based PGx assays, our test exhibited performance levels closely aligned with the reported literature standards for accuracy (ranging from 93% to 100%) and precision (ranging from 97% to 100%) (Hartshorne et al., 2013; Martins et al., 2013; Borobia et al., 2018; Collins et al., 2019; Tang et al., 2021; Kanji et al., 2023).

While our study provides an in-depth analysis of the Illumina GSA chip for PGx testing, it is also important to address its limitations. Firstly, our validation, conducted using cell lines, highlights the need for further research with clinically relevant samples before deployment in healthcare settings, as these samples would more accurately represent the complexity of genetic variation and its impact on drug metabolism. Secondly, using microarrays as the genotyping platform presents specific challenges. For instance, complex genetic events, particularly in genes like *CYP2D6*, are difficult to accurately genotype due to structural variants, hybrid rearrangements, and pseudogenes. In our assessment of SV calling performance for *CYP2D6*, we noted that no signature of a duplication was detected except for one instance of a non-identical whole gene duplication (NA18526, *1/*36 × 2+*10). In general, such hybrid genes are detected only over specific exon/introns depending on whether the tandem duplication occurs over the 3′ or the 5′ end of the gene. Based on the current settings, the minimum size of a reported CNV is 250 bp, which may be too small to identify most of such duplications. Potentially, reducing the minimum CNV size may allow better detection of such variations, however, it is expected that a higher number of false positives may also be reported. Additionally, diplotype concordance for SV-containing star-alleles in *CYP2D6* was lower than that of the other evaluated diplotypes (91.30% if ignoring tandem hybrid samples vs 87.50% for all samples). Similarly, we encountered limitations in detecting repeats in *UGT1A1*. These observations led us to exclude this specific variant type from the reportable range of the assay, a decision aligned with common practices in the field, including the Association for Molecular Pathology’s guidelines for PGx testing (Pratt et al., 2018; Pratt et al., 2019; Pratt et al., 2021). However, it does mean that not all possible diplotypes are captured by the assay. Another limitation of microarrays is their restricted ability to fully phase variant calls, which has affected our resolution of calls for genes such as *CYP2C9*. Furthermore, when specific markers are missing on the chip, or signal does not overlap with the expected clusters, we rely on imputation, limiting our ability to detect rare or novel alleles, as observed in our variant calls for *SLCO1B1* and *CYP2C19*. Emerging sequencing technologies, especially long-read sequencing, are poised to bridge this performance gap and standardize reportable loci, thereby minimizing discrepancies across tests (van der Lee et al., 2020). However, these methods are comparatively more expensive and are typically reserved for situations where cost-efficiency is not the primary consideration. Targeted assays that could reduce the costs of long-read sequencing are beginning to emerge (e.g., Twist Alliance Long-Read PGx Panel), but they still require significant upfront investment in the instrument. Therefore, our primary goal in this study was to develop a pharmacogenomics test with broad adoption potential, leading us to choose the Illumina GSA platform for its widespread availability and cost-effectiveness. Furthermore, the platform’s capacity to provide data beyond the target loci of the PGx assay opens the door to various additional applications.

Altogether, the notion of pre-emptive PGx testing seamlessly integrating into healthcare frameworks is no longer a distant vision but an impending reality. Numerous research endeavors, including randomized controlled trials, have gathered evidence supporting the customization of drug therapy based on pharmacogenetic testing targeting specific drug-gene interactions to improve patient outcomes (Mallal et al., 2008; Pirmohamed et al., 2013; Coenen et al., 2015; Henricks et al., 2018; Claassens et al., 2019). Additionally, several studies have reported significant reductions in hospital admissions, emergency department visits, and overall healthcare expenditures, indicating the potential cost-effectiveness of genetics-informed treatment approaches (Brixner et al., 2016; Finkelstein et al., 2016; Elliott et al., 2017). Notably, the PREPARE study, conducted across seven European countries with diverse healthcare settings and encompassing a cohort of 6,944 patients, evaluated the impact of genotype-guided prescriptions using a 12-gene pharmacogenetic panel (Swen et al., 2023). This prospective real-world implementation study revealed a 30% reduction in clinically relevant adverse drug reactions when employing a panel-based pharmacogenetic testing strategy.

However, the universal integration of PGx into the standard healthcare landscape is not without its challenges. These encompass a range of practical considerations that extend beyond the accuracy of the underlying genetic tests (Pirmohamed, 2023). Among them, the notable lack of awareness and training among healthcare professionals often leads to suboptimal utilization of pharmacogenomic testing. This knowledge gap extends beyond healthcare practitioners to include patients, who frequently remain uninformed about the potential benefits and accessibility of such tests. Operational challenges are also present and entail the need for streamlined processes for test ordering, result interpretation, and the immediate availability of results to healthcare professionals during patient care. Furthermore, the financial aspect should not be underestimated, particularly in countries lacking uniform insurance coverage, where the cost implications of pharmacogenomic testing can be prohibitive, even with cost-efficient solutions like microarrays. Lastly, navigating the ethical and privacy dilemmas associated with issues such as informed consent, data confidentiality, and the potential for genetic discrimination adds further layers of complexity. Addressing these intricate challenges requires a collaborative effort involving researchers, healthcare experts, policymakers, educators, and various stakeholders. Based on our research, which leverages an easily accessible microarray chip, we maintain an optimistic outlook that our solution, alongside others, will serve as a catalyst for the broader adoption of PGx testing.

## Data Availability

The original contributions presented in the study are publicly available. This data can be found here: The European Genome-Phenome Archive (https://ega.crg.eu/). Accession Number EGAS00001007710.

## Abbreviations

1KGP, 1000 Genomes Project; ADR, adverse drug reactions; BAF, B-allele frequency; CDC, United States Centers for Disease Control and Prevention; CNV, copy number variations; GIAB, Genome In a Bottle; GSA, global screening array; GeT-RM, Genetic Testing Reference Material; PGx, pharmacogenomics; CPIC, Clinical Pharmacogenetics Implementation Consortium; DPWG, Dutch Pharmacogenetics Working Group, MAF, minor allele frequency; SNP, single nucleotide polymorphism; CHS, Southern Han Chinese; CDX, Chinese Dai in Xishuangbanna, China; KHV, Kinh in Vietnam; CHB, Han Chinese in Beijing; JPT, Japanese people in Tokyo; LRR, log normalized intensity values ratio; PFB, Population frequency of B allele; CYP, cytochrome P450; MT-RNR1, Mitochondrially Encoded 12S RRNA; NUDT15, Nudix Hydrolase 15; DPYD, Dihydropyrimidine Dehydrogenase; G6PD, Glucose-6-Phosphate Dehydrogenase; UGT1A1, UDP Glucuronosyltransferase Family 1 Member A1; SLCO1B1, Solute Carrier Organic Anion Transporter Family Member 1B1; TPMT, Thiopurine S-methyltransferase.
