# Supplementary material for: Development and validation of a pharmacogenomics reporting workflow based on the illumina global screening array chip
Source: Front Pharmacol. 2024 Mar 11;15:1349203. doi: 10.3389/fphar.2024.1349203 (PMC10961362; doi:10.3389/fphar.2024.1349203)
Supplement: Supplementary file 1 [file Table1.DOCX]

**Supplementary Table 1: Details of the 503 variants reported**

| Chr | Position | | rsID | Associated gene | Associated allele | Is imputed |
| --- | --- | --- | --- | --- | --- | --- |
| chr1 | 97078987 | | rs114096998 | *DPYD* | c.3067C>A | 0 |
| chr1 | 97078993 | | rs148799944 | *DPYD* | c.3061G>C | 0 |
| chr1 | 97079005 | | rs140114515 | *DPYD* | c.3049G>A | 0 |
| chr1 | 97079071 | | rs1801268 | *DPYD* | c.2983G>T(*10) | 0 |
| chr1 | 97079076 | | rs139459586 | *DPYD* | c.2978T>G | 0 |
| chr1 | 97079077 | | rs202144771 | *DPYD* | c.2977C>T | 0 |
| chr1 | 97079121 | | rs72547601 | *DPYD* | c.2933A>G | 0 |
| chr1 | 97079133 | | rs72547602 | *DPYD* | c.2921A>T | 0 |
| chr1 | 97079139 | | rs145529148 | *DPYD* | c.2915A>G | 0 |
| chr1 | 97082365 | | rs141044036 | *DPYD* | c.2872A>G | 0 |
| chr1 | 97082391 | | rs67376798 | *DPYD* | c.2846A>T | 0 |
| chr1 | 97098598 | | rs1801267 | *DPYD* | c.2657G>A(*9B) | 0 |
| chr1 | 97098599 | | rs147545709 | *DPYD* | c.2656C>T | 0 |
| chr1 | 97098616 | | rs55674432 | *DPYD* | c.2639G>T | 0 |
| chr1 | 97098632 | | rs201035051 | *DPYD* | c.2623A>C | 0 |
| chr1 | 97193109 | | rs60139309 | *DPYD* | c.2582A>G | 0 |
| chr1 | 97193209 | | rs200687447 | *DPYD* | c.2482G>A | 0 |
| chr1 | 97234958 | | rs199634007 | *DPYD* | c.2336C>A | 0 |
| chr1 | 97305279 | | rs112766203 | *DPYD* | c.2279C>T | 0 |
| chr1 | 97305363 | | rs60511679 | *DPYD* | c.2195T>G | 0 |
| chr1 | 97305364 | | rs1801160 | *DPYD* | c.2194G>A(*6) | 0 |
| chr1 | 97305372 | | rs146529561 | *DPYD* | c.2186C>T | 0 |
| chr1 | 97306195 | | rs145548112 | *DPYD* | c.2161G>A | 0 |
| chr1 | 97373598 | | rs137999090 | *DPYD* | c.2021G>A | 0 |
| chr1 | 97373629 | | rs138545885 | *DPYD* | c.1990G>T | 0 |
| chr1 | 97382461 | | rs55971861 | *DPYD* | c.1906A>C | 0 |
| chr1 | 97450058 | | rs3918290 | *DPYD* | c.1905+1G>A(*2A) | 0 |
| chr1 | 97450065 | | rs72549303 | *DPYD* | c.1898delC(*3) | 0 |
| chr1 | 97450068 | | rs17376848 | *DPYD* | c.1896T>C | 0 |
| chr1 | 97450168 | | rs147601618 | *DPYD* | c.1796T>C | 0 |
| chr1 | 97450187 | | rs145773863 | *DPYD* | c.1777G>A | 0 |
| chr1 | 97450189 | | rs138616379 | *DPYD* | c.1775G>A | 0 |
| chr1 | 97515784 | | rs201615754 | *DPYD* | c.1682G>T | 0 |
| chr1 | 97515787 | | rs55886062 | *DPYD* | c.1679T>G(*13) | 0 |
| chr1 | 97515839 | | rs1801159 | *DPYD* | c.1627A>G(*5) | 0 |
| chr1 | 97515851 | | rs142619737 | *DPYD* | c.1615G>A | 0 |
| chr1 | 97515865 | | rs1801158 | *DPYD* | c.1601G>A(*4) | 0 |
| chr1 | 97515889 | rs190951787 | | *DPYD* | c.1577C>G | 0 |
| chr1 | 97515923 | rs148994843 | | *DPYD* | c.1543G>A | 0 |
| chr1 | 97549565 | rs138391898 | | *DPYD* | c.1519G>A | 0 |
| chr1 | 97549600 | rs111858276 | | *DPYD* | c.1484A>G | 0 |
| chr1 | 97549609 | rs72549304 | | *DPYD* | c.1475C>T | 0 |
| chr1 | 97549681 | rs199549923 | | *DPYD* | c.1403C>A | 0 |
| chr1 | 97549726 | rs144395748 | | *DPYD* | c.1358C>G | 0 |
| chr1 | 97549735 | rs72975710 | | *DPYD* | c.1349C>T | 0 |
| chr1 | 97573785 | rs186169810 | | *DPYD* | c.1314T>G | 0 |
| chr1 | 97573805 | rs142512579 | | *DPYD* | c.1294G>A | 0 |
| chr1 | 97573839 | rs200064537 | | *DPYD* | c.1260T>A | 0 |
| chr1 | 97573863 | rs56038477 | | *DPYD* | c.1129-5923C>G, c.1236G>A (HapB3) | 0 |
| chr1 | 97573881 | rs61622928 | | *DPYD* | c.1218G>A | 0 |
| chr1 | 97573918 | rs143815742 | | *DPYD* | c.1181G>T | 0 |
| chr1 | 97573919 | rs140602333 | | *DPYD* | c.1180C>T | 0 |
| chr1 | 97573943 | rs78060119 | | *DPYD* | c.1156G>T(*12) | 0 |
| chr1 | 97579893 | rs75017182 | | *DPYD* | c.1129-5923C>G, c.1236G>A (HapB3) | 0 |
| chr1 | 97593238 | rs72549305 | | *DPYD* | c.1108A>G | 0 |
| chr1 | 97593289 | rs143154602 | | *DPYD* | c.1057C>T | 0 |
| chr1 | 97593343 | rs72549306 | | *DPYD* | c.1003G>T(*11) | 0 |
| chr1 | 97593379 | rs201018345 | | *DPYD* | c.967G>A | 0 |
| chr1 | 97595083 | rs145112791 | | *DPYD* | c.934C>T | 0 |
| chr1 | 97595088 | rs150437414 | | *DPYD* | c.929T>C | 0 |
| chr1 | 97595149 | rs146356975 | | *DPYD* | c.868A>G | 0 |
| chr1 | 97679170 | rs45589337 | | *DPYD* | c.775A>G | 0 |
| chr1 | 97691776 | rs1801266 | | *DPYD* | c.703C>T(*8) | 0 |
| chr1 | 97699399 | rs72549307 | | *DPYD* | c.632A>G | 0 |
| chr1 | 97699430 | rs72549308 | | *DPYD* | c.601A>C | 0 |
| chr1 | 97699474 | rs115232898 | | *DPYD* | c.557A>G | 0 |
| chr1 | 97699533 | rs139834141 | | *DPYD* | c.498G>A | 0 |
| chr1 | 97699535 | rs2297595 | | *DPYD* | c.496A>G | 0 |
| chr1 | 97721542 | rs200562975 | | *DPYD* | c.451A>G | 0 |
| chr1 | 97740400 | rs150385342 | | *DPYD* | c.313G>A | 0 |
| chr1 | 97740410 | rs72549309 | | *DPYD* | c.295_298delTCAT(*7) | 0 |
| chr1 | 97883329 | rs1801265 | | *DPYD* | c.85T>C(*9A) | 0 |
| chr1 | 97883352 | rs80081766 | | *DPYD* | c.62G>A | 0 |
| chr1 | 97883353 | rs72549310 | | *DPYD* | c.61C>T | 0 |
| chr1 | 97883368 | rs150036960 | | *DPYD* | c.46C>G | 0 |
| chr1 | 201060815 | rs1800559 | | *CACNA1S* |  | 0 |
| chr1 | 201091993 | rs772226819 | | *CACNA1S* |  | 0 |
| chr10 | 94645745 | rs12777823 | | *.* |  | 0 |
| chr10 | 94761900 | rs12248560 | | *CYP2C19* | *1;*4;*17 | 1 |
| chr10 | 94762706 | rs28399504 | | *CYP2C19* | *1;*4 | 0 |
| chr10 | 94762755 | rs55752064 | | *CYP2C19* | *1;*14 | 0 |
| chr10 | 94762788 | rs1564656981 | | *CYP2C19* | *1;*29 | 0 |
| chr10 | 94762856 | rs1564657013 | | *CYP2C19* | *1;*19 | 0 |
| chr10 | 94775106 | rs145328984 | | *CYP2C19* | *1;*30 | 0 |
| chr10 | 94775121 | rs1564660997 | | *CYP2C19* | *1;*31 | 0 |
| chr10 | 94775160 | rs118203756 | | *CYP2C19* | *1;*23 | 0 |
| chr10 | 94775185 | rs1288601658 | | *CYP2C19* | *1;*32 | 0 |
| chr10 | 94775367 | rs12769205 | | *CYP2C19* | *1;*2;*35 | 0 |
| chr10 | 94775416 | rs41291556 | | *CYP2C19* | *1;*8 | 0 |
| chr10 | 94775423 | rs17885179 | | *CYP2C19* | *1;*39 | 0 |
| chr10 | 94775453 | rs72552267 | | *CYP2C19* | *1;*6 | 0 |
| chr10 | 94780574 | rs140278421 | | *CYP2C19* | *1;*22 | 0 |
| chr10 | 94780653 | rs4986893 | | *CYP2C19* | *1;*3 | 0 |
| chr10 | 94781858 | rs6413438 | | *CYP2C19* | *1;*10 | 0 |
| chr10 | 94781859 | rs4244285 | | *CYP2C19* | *1;*2 | 0 |
| chr10 | 94781944 | rs375781227 | | *CYP2C19* | *1;*26 | 0 |
| chr10 | 94781999 | rs72558186 | | *CYP2C19* | *1;*7 | 0 |
| chr10 | 94842861 | rs138142612 | | *CYP2C19* | *1;*18 | 0 |
| chr10 | 94842866 | rs3758581 | | *CYP2C19* | *1;*2;*3;*4;*5;*6;*7;*8;*9;*10;*11;*12;*13;*14;*15;*17;*18;*19;*22;*23;*24;*25;*26;*28;*29;*31;*32;*33;*35;*39 | 1 |
| chr10 | 94849995 | rs17879685 | | *CYP2C19* | *1;*13 | 0 |
| chr10 | 94852738 | rs56337013 | | *CYP2C19* | *1;*5 | 0 |
| chr10 | 94852785 | rs118203759 | | *CYP2C19* | *1;*25 | 0 |
| chr10 | 94852914 | rs55640102 | | *CYP2C19* | *1;*12 | 0 |
| chr10 | 94938683 | rs114071557 | | *CYP2C9* | *36 | 0 |
| chr10 | 94938771 | rs142240658 | | *CYP2C9* | *21 | 0 |
| chr10 | 94938803 | rs2031308986 | | *CYP2C9* | *22 | 0 |
| chr10 | 94938828 | rs564813580 | | *CYP2C9* | *37 | 0 |
| chr10 | 94941897 | rs371055887 | | *CYP2C9* | *20 | 0 |
| chr10 | 94941958 | rs72558187 | | *CYP2C9* | *13 | 0 |
| chr10 | 94941976 | chr10:94941976:G:C | | *CYP2C9* | *38 | 0 |
| chr10 | 94941982 | rs762239445 | | *CYP2C9* | *39 | 0 |
| chr10 | 94942018 | chr10:94942018:T:C | | *CYP2C9* | *40 | 0 |
| chr10 | 94942230 | rs767576260 | | *CYP2C9* | *43 | 0 |
| chr10 | 94942231 | rs12414460 | | *CYP2C9* | *42 | 0 |
| chr10 | 94942234 | rs72558189 | | *CYP2C9* | *14;*35 | 0 |
| chr10 | 94942249 | rs200965026 | | *CYP2C9* | *26;*44 | 0 |
| chr10 | 94942254 | rs199523631 | | *CYP2C9* | *45 | 0 |
| chr10 | 94942255 | rs200183364 | | *CYP2C9* | *33 | 0 |
| chr10 | 94942290 | rs1799853 | | *CYP2C9* | *2;*35;*61 | 0 |
| chr10 | 94942305 | rs754487195 | | *CYP2C9* | *46 | 0 |
| chr10 | 94942309 | rs7900194 | | *CYP2C9* | *8;*27 | 0 |
| chr10 | 94947782 | rs72558190 | | *CYP2C9* | *15 | 0 |
| chr10 | 94947785 | rs774550549 | | *CYP2C9* | *47 | 0 |
| chr10 | 94947907 | chr10:94947907:A:C | | *CYP2C9* | *57 | 0 |
| chr10 | 94947917 | rs1326630788 | | *CYP2C9* | *48 | 0 |
| chr10 | 94949129 | chr10:94949129:A:G | | *CYP2C9* | *49 | 0 |
| chr10 | 94949144 | chr10:94949144:C:T | | *CYP2C9* | *50 | 0 |
| chr10 | 94949217 | rs2256871 | | *CYP2C9* | *9 | 0 |
| chr10 | 94949280 | rs9332130 | | *CYP2C9* | *10;*71 | 0 |
| chr10 | 94949281 | rs9332131 | | *CYP2C9* | *6 | 0 |
| chr10 | 94972119 | rs182132442 | | *CYP2C9* | *29 | 0 |
| chr10 | 94972134 | chr10:94972134:A:G | | *CYP2C9* | *51 | 0 |
| chr10 | 94972179 | rs72558192 | | *CYP2C9* | *16 | 0 |
| chr10 | 94972180 | rs988617574 | | *CYP2C9* | *52 | 0 |
| chr10 | 94972233 | rs1237225311 | | *CYP2C9* | *53 | 0 |
| chr10 | 94981201 | rs57505750 | | *CYP2C9* | *31 | 0 |
| chr10 | 94981224 | rs28371685 | | *CYP2C9* | *11 | 0 |
| chr10 | 94981250 | rs750820937 | | *CYP2C9* | *54 | 0 |
| chr10 | 94981281 | rs749060448 | | *CYP2C9* | *24 | 0 |
| chr10 | 94981296 | rs1057910 | | *CYP2C9* | *3;*18;*68 | 0 |
| chr10 | 94981297 | rs56165452 | | *CYP2C9* | *4 | 0 |
| chr10 | 94981301 | rs28371686 | | *CYP2C9* | *5 | 0 |
| chr10 | 94981302 | rs1250577724 | | *CYP2C9* | *55 | 0 |
| chr10 | 94981305 | rs578144976 | | *CYP2C9* | *66 | 0 |
| chr10 | 94981365 | chr10:94981365:C:T | | *CYP2C9* | *17 | 0 |
| chr10 | 94986042 | rs764211126 | | *CYP2C9* | *56 | 0 |
| chr10 | 94986073 | rs72558193 | | *CYP2C9* | *18 | 0 |
| chr10 | 94988917 | rs769942899 | | *CYP2C9* | *19 | 0 |
| chr10 | 94988955 | rs767284820 | | *CYP2C9* | *60 | 0 |
| chr10 | 94988984 | rs781583846 | | *CYP2C9* | *30 | 0 |
| chr10 | 94989020 | rs9332239 | | *CYP2C9* | *12;*71 | 0 |
| chr10 | 94989023 | rs868182778 | | *CYP2C9* | *32 | 0 |
| chr10 | 95038992 | rs10509681 | | *CYP2C8* |  | 0 |
| chr10 | 95042890 | chr10:95042890:C:A | | *CYP2C8* |  | 0 |
| chr10 | 95042958 | rs45438799 | | *CYP2C8* |  | 0 |
| chr10 | 95045951 | rs78637571 | | *CYP2C8* |  | 0 |
| chr10 | 95058349 | rs11572103 | | *CYP2C8* |  | 0 |
| chr10 | 95058362 | rs1058930 | | *CYP2C8* |  | 0 |
| chr10 | 95058414 | rs769460274 | | *CYP2C8* |  | 0 |
| chr10 | 95058485 | chr10:95058485:A:C | | *CYP2C8* |  | 0 |
| chr10 | 95064886 | rs72558195 | | *CYP2C8* |  | 0 |
| chr10 | 95064901 | rs41286886 | | *CYP2C8* |  | 0 |
| chr10 | 95064931 | rs142886225 | | *CYP2C8* |  | 1 |
| chr10 | 95067273 | rs11572080 | | *CYP2C8* |  | 0 |
| chr10 | 95069673 | rs7909236 | | *CYP2C8* |  | 1 |
| chr10 | 95069772 | rs17110453 | | *CYP2C8* |  | 1 |
| chr12 | 21172734 | rs139257324 | | *SLCO1B1* | *33 | 0 |
| chr12 | 21172776 | rs373327528 | | *SLCO1B1* | *23 | 0 |
| chr12 | 21172782 | rs56101265 | | *SLCO1B1* | *2;*12 | 0 |
| chr12 | 21174595 | rs56061388 | | *SLCO1B1* | *3;*13 | 0 |
| chr12 | 21176804 | rs2306283 | | *SLCO1B1* | *14;*15;*20;*24;*25;*27;*28;*29;*30;*31;*32;*33;*37;*39;*42;*43;*44;*46;*47 | 0 |
| chr12 | 21176868 | rs2306282 | | *SLCO1B1* | *16 | 0 |
| chr12 | 21176879 | rs11045819 | | *SLCO1B1* | *4;*14;*25;*32;*43 | 1 |
| chr12 | 21176883 | rs72559745 | | *SLCO1B1* | *3;*13 | 0 |
| chr12 | 21176898 | rs77271279 | | *SLCO1B1* | *41 | 0 |
| chr12 | 21178615 | rs4149056 | | *SLCO1B1* | *5;*15;*40;*46;*47 | 0 |
| chr12 | 21178957 | rs79135870 | | *SLCO1B1* | *30 | 0 |
| chr12 | 21196951 | rs11045852 | | *SLCO1B1* | *24;*25;*28;*32;*33;*43;*44 | 0 |
| chr12 | 21196976 | rs11045853 | | *SLCO1B1* | *25;*28;*33 | 0 |
| chr12 | 21200544 | rs72559747 | | *SLCO1B1* | *47 | 0 |
| chr12 | 21200595 | rs55901008 | | *SLCO1B1* | *6 | 0 |
| chr12 | 21202555 | rs59113707 | | *SLCO1B1* | *27 | 0 |
| chr12 | 21202649 | rs56387224 | | *SLCO1B1* | *7 | 0 |
| chr12 | 21202664 | rs142965323 | | *SLCO1B1* | *26 | 0 |
| chr12 | 21205921 | rs72559748 | | *SLCO1B1* | *8 | 0 |
| chr12 | 21205999 | rs59502379 | | *SLCO1B1* | *9;*31 | 0 |
| chr12 | 21206031 | rs74064213 | | *SLCO1B1* | *43;*44 | 1 |
| chr12 | 21222355 | rs71581941 | | *SLCO1B1* | *45;*46 | 0 |
| chr12 | 21239042 | rs34671512 | | *SLCO1B1* | *19;*20;*40 | 0 |
| chr12 | 21239077 | rs56199088 | | *SLCO1B1* | *10;*12 | 0 |
| chr12 | 21239113 | rs55737008 | | *SLCO1B1* | *11;*13 | 0 |
| chr12 | 21239145 | rs200995543 | | *SLCO1B1* | *34 | 0 |
| chr12 | 21239158 | rs140790673 | | *SLCO1B1* | *29 | 0 |
| chr13 | 48037782 | rs746071566 | | *NUDT15* | *2;*6;*9 | 1 |
| chr13 | 48037798 | rs186364861 | | *NUDT15* | *5 | 0 |
| chr13 | 48045719 | rs116855232 | | *NUDT15* | *2;*3 | 0 |
| chr13 | 48045720 | rs147390019 | | *NUDT15* | *4 | 0 |
| chr15 | 74745879 | rs2069514 | | *.* |  | 1 |
| chr16 | 31096367 | rs9923231 | | *.* |  | 1 |
| chr16 | 31096368 | rs9923231 | | *VKORC1* |  | 0 |
| chr19 | 15879621 | rs2108622 | | *CYP4F2* | *3 | 0 |
| chr19 | 15897578 | rs3093105 | | *CYP4F2* | *2 | 1 |
| chr19 | 38440802 | rs193922747 | | *RYR1* | c.103T>C | 0 |
| chr19 | 38440829 | rs193922748 | | *RYR1* | c.130C>T | 0 |
| chr19 | 38444211 | rs118192161 | | *RYR1* | c.487C>T | 0 |
| chr19 | 38444212 | rs193922753 | | *RYR1* | c.488G>T | 0 |
| chr19 | 38446710 | rs1801086 | | *RYR1* | c.742G>A;c.742G>C | 0 |
| chr19 | 38448712 | rs121918592 | | *RYR1* | c.1021G>A;c.1021G>C | 0 |
| chr19 | 38451842 | rs193922764 | | *RYR1* | c.1201C>T | 0 |
| chr19 | 38451850 | rs118192116 | | *RYR1* | c.1209C>G | 0 |
| chr19 | 38455359 | rs118192162 | | *RYR1* | c.1565A>C | 0 |
| chr19 | 38455463 | rs111888148 | | *RYR1* | c.1589G>A | 0 |
| chr19 | 38455471 | rs193922768 | | *RYR1* | c.1597C>T | 0 |
| chr19 | 38455528 | rs193922770 | | *RYR1* | c.1654C>T | 0 |
| chr19 | 38457545 | rs118192172 | | *RYR1* | c.1840C>T | 0 |
| chr19 | 38457546 | rs193922772 | | *RYR1* | c.1841G>T | 0 |
| chr19 | 38494564 | rs118192175 | | *RYR1* | c.6487C>T | 0 |
| chr19 | 38494579 | rs118192176 | | *RYR1* | c.6502G>A | 0 |
| chr19 | 38496283 | rs118192177 | | *RYR1* | c.6617C>G;c.6617C>T | 0 |
| chr19 | 38499223 | rs112563513 | | *RYR1* | c.7007G>A | 0 |
| chr19 | 38499655 | rs193922802 | | *RYR1* | c.7048G>A | 0 |
| chr19 | 38499670 | rs193922803 | | *RYR1* | c.7063C>T | 0 |
| chr19 | 38499731 | rs193922807 | | *RYR1* | c.7124G>C | 0 |
| chr19 | 38499975 | rs193922809 | | *RYR1* | c.7282G>A | 0 |
| chr19 | 38499993 | rs121918593 | | *RYR1* | c.7300G>A | 0 |
| chr19 | 38499997 | rs28933396 | | *RYR1* | c.7304G>A | 0 |
| chr19 | 38500636 | rs118192124 | | *RYR1* | c.7354C>T | 0 |
| chr19 | 38500642 | rs193922816 | | *RYR1* | c.7360C>T | 0 |
| chr19 | 38500643 | rs118192122 | | *RYR1* | c.7361G>A | 0 |
| chr19 | 38500654 | rs28933397 | | *RYR1* | c.7372C>T | 0 |
| chr19 | 38500655 | rs121918594 | | *RYR1* | c.7373G>A | 0 |
| chr19 | 38500898 | rs118192178 | | *RYR1* | c.7522C>G;c.7522C>T | 0 |
| chr19 | 38500899 | rs193922818 | | *RYR1* | c.7523G>A | 0 |
| chr19 | 38512321 | rs193922832 | | *RYR1* | c.9310G>A | 0 |
| chr19 | 38543832 | rs193922843 | | *RYR1* | c.11969G>T | 0 |
| chr19 | 38580004 | rs118192167 | | *RYR1* | c.14387A>G | 0 |
| chr19 | 38580094 | rs121918595 | | *RYR1* | c.14477C>T | 0 |
| chr19 | 38580114 | rs193922876 | | *RYR1* | c.14497C>T | 0 |
| chr19 | 38580370 | rs193922878 | | *RYR1* | c.14512C>G | 0 |
| chr19 | 38580403 | rs118192168 | | *RYR1* | c.14545G>A | 0 |
| chr19 | 38580440 | rs63749869 | | *RYR1* | c.14582G>A | 0 |
| chr19 | 38584989 | rs118192170 | | *RYR1* | c.14693T>C | 0 |
| chr19 | 39248147 | rs12979860 | | *IFNL3* |  | 1 |
| chr19 | 40991224 | rs34223104 | | *CYP2B6* | *22;*34;*35;*36 | 1 |
| chr19 | 40991369 | rs8192709 | | *CYP2B6* | *2;*10 | 0 |
| chr19 | 40991388 | rs33980385 | | *CYP2B6* | *17 | 0 |
| chr19 | 40991441 | rs35303484 | | *CYP2B6* | *11 | 0 |
| chr19 | 41004125 | rs36060847 | | *CYP2B6* | *12 | 0 |
| chr19 | 41004158 | rs186335453 | | *CYP2B6* | *35 | 0 |
| chr19 | 41004377 | rs12721655 | | *CYP2B6* | *8;*13 | 0 |
| chr19 | 41004406 | rs145884402 | | *CYP2B6* | *35 | 0 |
| chr19 | 41006919 | rs3826711 | | *CYP2B6* | *26 | 1 |
| chr19 | 41006923 | rs36056539 | | *CYP2B6* | *20 | 0 |
| chr19 | 41006936 | rs3745274 | | *CYP2B6* | *6;*7;*9;*13;*19;*20;*26;*34;*36;*37;*38 | 0 |
| chr19 | 41006968 | rs373489637 | | *CYP2B6* | *37 | 0 |
| chr19 | 41007013 | rs36079186 | | *CYP2B6* | *27;*35 | 0 |
| chr19 | 41009350 | rs45482602 | | *CYP2B6* | *3 | 0 |
| chr19 | 41010006 | rs139029625 | | *CYP2B6* | *35 | 0 |
| chr19 | 41010088 | rs34698757 | | *CYP2B6* | *28 | 0 |
| chr19 | 41012316 | rs28399499 | | *CYP2B6* | *18 | 0 |
| chr19 | 41012339 | rs34826503 | | *CYP2B6* | *19 | 0 |
| chr19 | 41012693 | rs35979566 | | *CYP2B6* | *15 | 0 |
| chr19 | 41012803 | rs35010098 | | *CYP2B6* | *21 | 0 |
| chr19 | 41016726 | rs3211369 | | *CYP2B6* | *23 | 1 |
| chr2 | 233759924 | rs887829 | | *UGT1A1* | *80;*80+*28;*80+*37 | 1 |
| chr2 | 233760498 | rs4148323 | | *UGT1A1* | *6 | 0 |
| chr2 | 233760973 | rs35350960 | | *UGT1A1* | *27 | 0 |
| chr22 | 19963748 | rs4680 | | *.* |  | 0 |
| chr22 | 42126611 | rs1135840 | | *CYP2D6* | *2;*4;*6;*8;*10;*11;*12;*14;*17;*19;*20;*21;*28;*29;*30;*31;*32;*35;*36;*37;*39;*40;*41;*42;*45;*46;*47;*49;*51;*52;*54;*55;*56;*57;*58;*59;*64;*65;*69;*70;*72;*73;*83;*84;*85;*87;*88;*94;*95;*98;*99;*100;*101;*102;*103;*104;*105;*111;*114;*117;*121;*123;*125;*126;*128;*129;*132;*133;*135;*136;*138;*141;*142;*146;*147;*148;*149;*150;*154;*155;*156;*157;*158;*159;*160;*161;*162;*163;*164;*165;*166;*171 | 1 |
| chr22 | 42126623 | rs75467367 | | *CYP2D6* | *4;*36;*57;*83;*141 | 0 |
| chr22 | 42126624 | rs74478221 | | *CYP2D6* | *4;*36;*57;*83;*141 | 0 |
| chr22 | 42126627 | chr22:42126627:A:C | | *CYP2D6* | *4;*36;*57;*83;*141 | 0 |
| chr22 | 42126635 | rs766507177 | | *CYP2D6* | *4;*36;*57;*83;*141 | 0 |
| chr22 | 42126636 | rs28371735 | | *CYP2D6* | *4;*36;*57;*83;*141 | 0 |
| chr22 | 42126656 | rs765776661 | | *CYP2D6* | *18 | 0 |
| chr22 | 42126660 | rs1135835 | | *CYP2D6* | *4;*36;*57;*83;*141 | 0 |
| chr22 | 42126663 | rs1135833 | | *CYP2D6* | *4;*36;*57;*83;*141 | 0 |
| chr22 | 42126749 | rs267608319 | | *CYP2D6* | *31 | 1 |
| chr22 | 42126914 | rs28371733 | | *CYP2D6* | *52;*106 | 0 |
| chr22 | 42126956 | rs1931013246 | | *CYP2D6* | *55 | 0 |
| chr22 | 42127457 | rs77312092 | | *CYP2D6* | *95 | 0 |
| chr22 | 42127590 | rs72549347 | | *CYP2D6* | *56 | 0 |
| chr22 | 42127593 | rs267608295 | | *CYP2D6* | *25 | 0 |
| chr22 | 42127608 | rs59421388 | | *CYP2D6* | *29;*70;*109;*149;*155;*156;*157;*165;*171 | 0 |
| chr22 | 42127610 | rs748712690 | | *CYP2D6* | *94 | 0 |
| chr22 | 42127611 | rs78209835 | | *CYP2D6* | *117 | 0 |
| chr22 | 42127619 | rs72549348 | | *CYP2D6* | *51 | 0 |
| chr22 | 42127803 | rs28371725 | | *CYP2D6* | *32;*41;*69;*91;*119;*123;*138;*158 | 0 |
| chr22 | 42127841 | rs72549349 | | *CYP2D6* | *44 | 0 |
| chr22 | 42127845 | rs730882170 | | *CYP2D6* | *101 | 0 |
| chr22 | 42127852 | rs79292917 | | *CYP2D6* | *59 | 0 |
| chr22 | 42127856 | rs5030867 | | *CYP2D6* | *7 | 0 |
| chr22 | 42127941 | rs16947 | | *CYP2D6* | *2;*4;*8;*11;*12;*14;*17;*19;*20;*21;*28;*29;*30;*31;*32;*34;*35;*40;*41;*42;*45;*46;*51;*55;*56;*58;*59;*65;*69;*73;*84;*85;*91;*98;*102;*103;*104;*105;*111;*114;*117;*121;*123;*125;*126;*128;*129;*133;*135;*136;*138;*141;*146;*148;*149;*150;*154;*155;*156;*157;*158;*159;*160;*161;*162;*163;*165;*166;*171 | 1 |
| chr22 | 42127962 | rs267608279 | | *CYP2D6* | *100 | 0 |
| chr22 | 42127973 | rs1135829 | | *CYP2D6* | *115;*132 | 0 |
| chr22 | 42128173 | rs5030656 | | *CYP2D6* | *9;*109;*115 | 0 |
| chr22 | 42128211 | rs72549352 | | *CYP2D6* | *21 | 1 |
| chr22 | 42128212 | rs367543000 | | *CYP2D6* | *81 | 0 |
| chr22 | 42128235 | rs267608297 | | *CYP2D6* | *54 | 0 |
| chr22 | 42128241 | rs35742686 | | *CYP2D6* | *3 | 0 |
| chr22 | 42128248 | rs72549353 | | *CYP2D6* | *19 | 0 |
| chr22 | 42128308 | rs28371717 | | *CYP2D6* | *33 | 0 |
| chr22 | 42128807 | rs567606867 | | *CYP2D6* | *153 | 0 |
| chr22 | 42128814 | rs72549354 | | *CYP2D6* | *20 | 0 |
| chr22 | 42128932 | rs556882139 | | *CYP2D6* | *165 | 1 |
| chr22 | 42128945 | rs3892097 | | *CYP2D6* | *4 | 1 |
| chr22 | 42129033 | rs5030865 | | *CYP2D6* | *8;*14;*114 | 0 |
| chr22 | 42129042 | rs1135824 | | *CYP2D6* | *3;*103;*121 | 0 |
| chr22 | 42129071 | rs267608302 | | *CYP2D6* | *50;*104 | 0 |
| chr22 | 42129083 | rs5030655 | | *CYP2D6* | *6 | 0 |
| chr22 | 42129087 | rs78482768 | | *CYP2D6* | *28 | 0 |
| chr22 | 42129130 | rs1058164 | | *CYP2D6* | *2;*4;*8;*10;*11;*12;*14;*17;*19;*20;*21;*28;*29;*30;*31;*32;*35;*36;*37;*39;*40;*41;*42;*45;*46;*47;*49;*51;*52;*54;*55;*56;*57;*58;*59;*64;*65;*69;*70;*72;*73;*84;*85;*87;*88;*94;*95;*98;*99;*100;*101;*102;*103;*104;*105;*111;*117;*121;*123;*125;*126;*128;*129;*132;*133;*135;*136;*138;*141;*142;*146;*147;*148;*149;*150;*154;*155;*156;*157;*158;*159;*160;*161;*162;*163;*164;*165;*166;*171 | 1 |
| chr22 | 42129132 | rs61736512 | | *CYP2D6* | *29;*70;*107;*149;*155;*156;*157;*164;*165;*171 | 0 |
| chr22 | 42129180 | rs1135822 | | *CYP2D6* | *36;*49;*53 | 1 |
| chr22 | 42129759 | rs535642512 | | *CYP2D6* | *111 | 1 |
| chr22 | 42129765 | rs78459009 | | *CYP2D6* | *82 | 0 |
| chr22 | 42129770 | rs28371706 | | *CYP2D6* | *17;*40;*58;*64;*82;*141;*154 | 0 |
| chr22 | 42129771 | rs74802369 | | *CYP2D6* | *82 | 0 |
| chr22 | 42129779 | rs76187628 | | *CYP2D6* | *82;*88 | 0 |
| chr22 | 42129780 | rs267608308 | | *CYP2D6* | *73 | 0 |
| chr22 | 42129809 | rs28371704 | | *CYP2D6* | *4;*82;*160 | 1 |
| chr22 | 42129819 | rs28371703 | | *CYP2D6* | *4;*74;*82;*160 | 1 |
| chr22 | 42129821 | rs267608309 | | *CYP2D6* | *48;*102;*103 | 0 |
| chr22 | 42129827 | rs267608276 | | *CYP2D6* | *99 | 1 |
| chr22 | 42129836 | rs267608310 | | *CYP2D6* | *23 | 0 |
| chr22 | 42129910 | rs201377835 | | *CYP2D6* | *11 | 0 |
| chr22 | 42130667 | rs118203758 | | *CYP2D6* | *71;*168 | 0 |
| chr22 | 42130668 | rs5030862 | | *CYP2D6* | *12 | 0 |
| chr22 | 42130692 | rs1065852 | | *CYP2D6* | *4;*10;*36;*37;*47;*49;*52;*54;*56;*57;*64;*65;*69;*72;*87;*94;*95;*99;*100;*101;*114;*132;*142;*147;*150 | 1 |
| chr22 | 42130710 | rs138100349 | | *CYP2D6* | *22;*44;*142 | 0 |
| chr22 | 42130719 | rs267608313 | | *CYP2D6* | *47 | 0 |
| chr22 | 42130761 | rs769258 | | *CYP2D6* | *35;*143 | 1 |
| chr22 | 42130773 | rs72549358 | | *CYP2D6* | *28 | 0 |
| chr22 | 42130778 | rs773790593 | | *CYP2D6* | *87 | 0 |
| chr4 | 88131171 | rs2231142 | | *ABCG2* |  | 0 |
| chr6 | 18130687 | rs1142345 | | *TPMT* | *3A;*3C;*41 | 0 |
| chr6 | 18130694 | rs150900439 | | *TPMT* | *20 | 0 |
| chr6 | 18130725 | rs72552736 | | *TPMT* | *7 | 0 |
| chr6 | 18130758 | rs398122996 | | *TPMT* | *37 | 0 |
| chr6 | 18130762 | rs56161402 | | *TPMT* | *8 | 0 |
| chr6 | 18130772 | rs377085266 | | *TPMT* | *25 | 0 |
| chr6 | 18130781 | rs1800584 | | *TPMT* | *4 | 0 |
| chr6 | 18132136 | rs72556347 | | *TPMT* | *26 | 0 |
| chr6 | 18132147 | rs79901429 | | *TPMT* | *31 | 0 |
| chr6 | 18132163 | chr6:18132163:C:T | | *TPMT* | *36 | 0 |
| chr6 | 18133847 | rs6921269 | | *TPMT* | *24 | 0 |
| chr6 | 18133870 | rs772832951 | | *TPMT* | *38 | 0 |
| chr6 | 18133884 | rs74423290 | | *TPMT* | *23 | 0 |
| chr6 | 18133890 | rs9333570 | | *TPMT* | *15 | 0 |
| chr6 | 18138970 | rs112339338 | | *TPMT* | *33 | 0 |
| chr6 | 18138997 | rs1800460 | | *TPMT* | *3A;*3B | 0 |
| chr6 | 18139027 | rs72552737 | | *TPMT* | *10 | 0 |
| chr6 | 18139689 | rs72552738 | | *TPMT* | *11 | 0 |
| chr6 | 18143597 | chr6:18143597:T:G | | *TPMT* | *19 | 0 |
| chr6 | 18143606 | rs151149760 | | *TPMT* | *9 | 0 |
| chr6 | 18143613 | chr6:18143613:C:G | | *TPMT* | *28 | 0 |
| chr6 | 18143622 | rs115106679 | | *TPMT* | *32 | 0 |
| chr6 | 18143643 | chr6:18143643:A:C | | *TPMT* | *27 | 0 |
| chr6 | 18143718 | rs111901354 | | *TPMT* | *34 | 0 |
| chr6 | 18143724 | rs1800462 | | *TPMT* | *2 | 0 |
| chr6 | 18147838 | rs281874771 | | *TPMT* | *39 | 0 |
| chr6 | 18147845 | rs777686348 | | *TPMT* | *18 | 0 |
| chr6 | 18147851 | rs200591577 | | *TPMT* | *21 | 0 |
| chr6 | 18147856 | chr6:18147856:A:G | | *TPMT* | *35 | 0 |
| chr6 | 18147910 | rs72552740 | | *TPMT* | *5 | 0 |
| chr6 | 18149004 | chr6:18149004:G:C | | *TPMT* | *17 | 0 |
| chr6 | 18149022 | rs750424422 | | *TPMT* | *30 | 0 |
| chr6 | 18149045 | rs72552742 | | *TPMT* | *13 | 0 |
| chr6 | 18149126 | rs267607275 | | *TPMT* | *29 | 0 |
| chr6 | 18149127 | rs9333569 | | *TPMT* | *14 | 0 |
| chr6 | 154039662 | rs1799971 | | *.* |  | 0 |
| chr7 | 99652770 | rs41303343 | | *CYP3A5* | *7 | 0 |
| chr7 | 99660516 | rs28383479 | | *CYP3A5* | *9 | 0 |
| chr7 | 99665212 | rs10264272 | | *CYP3A5* | *6 | 0 |
| chr7 | 99672916 | rs776746 | | *CYP3A5* | *3 | 0 |
| chr7 | 99676198 | rs55817950 | | *CYP3A5* | *8 | 0 |
| chr7 | 99758183 | rs67666821 | | *CYP3A4* | *20 | 0 |
| chr7 | 99760901 | rs4986910 | | *CYP3A4* | *3;*37;*38 | 1 |
| chr7 | 99762047 | rs4986909 | | *CYP3A4* | *13 | 0 |
| chr7 | 99762177 | rs12721629 | | *CYP3A4* | *12 | 0 |
| chr7 | 99762206 | rs67784355 | | *CYP3A4* | *11;*38 | 0 |
| chr7 | 99763843 | rs2242480 | | *CYP3A4* |  | 0 |
| chr7 | 99763925 | rs201821708 | | *CYP3A4* | *21 | 0 |
| chr7 | 99764003 | rs28371759 | | *CYP3A4* | *18 | 0 |
| chr7 | 99766411 | rs4646438 | | *CYP3A4* | *6 | 0 |
| chr7 | 99766440 | rs138105638 | | *CYP3A4* | *26 | 0 |
| chr7 | 99768360 | rs55785340 | | *CYP3A4* | *2 | 0 |
| chr7 | 99768371 | rs55901263 | | *CYP3A4* | *5 | 0 |
| chr7 | 99768424 | rs113667357 | | *CYP3A4* | *24 | 0 |
| chr7 | 99768445 | rs4987159 | | *CYP3A4* |  | 1 |
| chr7 | 99768470 | rs12721627 | | *CYP3A4* | *16 | 0 |
| chr7 | 99768693 | rs35599367 | | *CYP3A4* | *22;*37 | 0 |
| chr7 | 99769769 | rs4986908 | | *CYP3A4* | *10 | 0 |
| chr7 | 99769781 | rs72552798 | | *CYP3A4* | *9 | 0 |
| chr7 | 99769804 | rs4986907 | | *CYP3A4* | *15 | 0 |
| chr7 | 99769805 | rs57409622 | | *CYP3A4* | *23 | 0 |
| chr7 | 99770165 | rs72552799 | | *CYP3A4* | *8 | 0 |
| chr7 | 99770202 | rs55951658 | | *CYP3A4* | *4 | 0 |
| chr7 | 99784038 | rs12721634 | | *CYP3A4* | *14 | 0 |
| chr7 | 99784237 | rs59537101 | | *CYP3A4* |  | 0 |
| chr7 | 99784473 | rs2740574 | | *CYP3A4* |  | 1 |
| chr7 | 117509035 | rs397508256 | | *CFTR* | E56K | 0 |
| chr7 | 117509069 | rs368505753 | | *CFTR* | P67L | 0 |
| chr7 | 117509089 | rs115545701 | | *CFTR* | R74W | 0 |
| chr7 | 117530953 | rs113993958 | | *CFTR* | D110H | 0 |
| chr7 | 117530955 | rs397508537 | | *CFTR* | D110E | 0 |
| chr7 | 117530974 | rs77834169 | | *CFTR* | R117C | 0 |
| chr7 | 117530975 | rs78655421 | | *CFTR* | R117H | 0 |
| chr7 | 117534318 | rs80282562 | | *CFTR* | G178R | 0 |
| chr7 | 117534368 | rs397508761 | | *CFTR* | 711+3A->G | 0 |
| chr7 | 117535285 | rs121908752 | | *CFTR* | L206W | 0 |
| chr7 | 117540270 | rs77932196 | | *CFTR* | R347H | 0 |
| chr7 | 117540285 | rs121908753 | | *CFTR* | R352Q | 0 |
| chr7 | 117548795 | rs74551128 | | *CFTR* | A455E | 0 |
| chr7 | 117559590 | rs113993960 | | *.* |  | 0 |
| chr7 | 117587799 | rs121908757 | | *CFTR* | S549R(A>C) | 0 |
| chr7 | 117587801 | rs121909005 | | *CFTR* | S549R(T>G) | 0 |
| chr7 | 117587805 | rs121909013 | | *CFTR* | G551S | 0 |
| chr7 | 117587806 | rs75527207 | | *CFTR* | G551D | 0 |
| chr7 | 117594930 | rs397508387 | | *CFTR* | E831X | 0 |
| chr7 | 117602868 | rs80224560 | | *CFTR* | 2789+5G->A | 0 |
| chr7 | 117603708 | rs397508442 | | *CFTR* | S945L | 0 |
| chr7 | 117606695 | rs141033578 | | *CFTR* | S977F | 0 |
| chr7 | 117611555 | rs76151804 | | *CFTR* | 3272-26A->G | 0 |
| chr7 | 117611595 | rs150212784 | | *CFTR* | F1052V | 0 |
| chr7 | 117611620 | rs397508513 | | *CFTR* | K1060T | 0 |
| chr7 | 117611640 | rs121909020 | | *CFTR* | A1067T | 0 |
| chr7 | 117611646 | rs200321110 | | *CFTR* | G1069R | 0 |
| chr7 | 117611649 | rs202179988 | | *CFTR* | R1070W | 0 |
| chr7 | 117611663 | rs186045772 | | *CFTR* | F1074L | 0 |
| chr7 | 117614699 | rs75541969 | | *CFTR* | D1152H | 0 |
| chr7 | 117639961 | rs75039782 | | *CFTR* | 3849+10kbC->T | 0 |
| chr7 | 117642472 | rs74503330 | | *CFTR* | S1251N | 0 |
| chr7 | 117642483 | rs121909041 | | *CFTR* | S1255P | 0 |
| chr7 | 117642528 | rs11971167 | | *CFTR* | D1270N | 0 |
| chr7 | 117664770 | rs193922525 | | *CFTR* | G1349D | 0 |
| chrMT | 663 | rs56489998 | | *MTRNR1* |  | 0 |
| chrMT | 827 | rs28358569 | | *MTRNR1* |  | 0 |
| chrMT | 951 | rs200887992 | | *MTRNR1* |  | 0 |
| chrMT | 1243 | rs28358572 | | *MTRNR1* |  | 0 |
| chrMT | 1553 | rs267606617 | | *MTRNR1* |  | 0 |
| chrX | 154532203 | rs137852348 | | *G6PD* | Split | 0 |
| chrX | 154532245 | rs137852344 | | *G6PD* | Neapolis | 0 |
| chrX | 154532257 | rs72554664 | | *G6PD* | Kaiping, Anant, Dhon, Sapporo-like, Wosera | 0 |
| chrX | 154532264 | rs782608284 | | *G6PD* | Yunan | 0 |
| chrX | 154532269 | rs72554665 | | *G6PD* | Bangkok Noi Canton, Taiwan-Hakka, Gifu-like, Agrigento-like Cosenza | 0 |
| chrX | 154532389 | rs137852324 | | *G6PD* | Andalus | 0 |
| chrX | 154532390 | rs398123546 | | *G6PD* | Hermoupolis Honiara Union,Maewo, Chinese-2, Kalo | 0 |
| chrX | 154532411 | rs137852317 | | *G6PD* | Santiago de Cuba, Morioka | 0 |
| chrX | 154532434 | rs137852337 | | *G6PD* | Pawnee | 0 |
| chrX | 154532439 | rs2230037 | | *G6PD* |  | 0 |
| chrX | 154532459 | rs782098548 | | *G6PD* | Surabaya | 0 |
| chrX | 154532625 | rs137852336 | | *G6PD* | Japan, Shinagawa Kawasaki | 0 |
| chrX | 154532626 | rs137852323 | | *G6PD* | Riverside | 0 |
| chrX | 154532662 | rs137852325 | | *G6PD* | Puerto Limon | 0 |
| chrX | 154532674 | rs137852335 | | *G6PD* | Alhambra | 0 |
| chrX | 154532676 | rs137852316 | | *G6PD* | Nashville, Anaheim, Portici | 0 |
| chrX | 154532694 | rs137852321 | | *G6PD* | Beverly Hills, Genova, Iwate, Niigata, Yamaguchi | 0 |
| chrX | 154532695 | rs137852334 | | *G6PD* | Guadalajara Mt Sinai | 0 |
| chrX | 154532698 | rs137852320 | | *G6PD* | Iowa, Walter Reed, Springfield | 0 |
| chrX | 154532701 | rs137852322 | | *G6PD* | Tomah | 0 |
| chrX | 154532722 | rs371489738 | | *G6PD* | Montpellier | 0 |
| chrX | 154532765 | rs137852329 | | *G6PD* | Aachen Loma Linda | 0 |
| chrX | 154532772 | rs137852345 | | *G6PD* | Serres | 0 |
| chrX | 154532797 | rs137852333 | | *G6PD* | Ierapetra | 0 |
| chrX | 154532956 | rs398123544 | | *G6PD* | Cincinnati | 0 |
| chrX | 154532969 | rs137852342 | | *G6PD* | Chinese-5 | 0 |
| chrX | 154532990 | rs5030869 | | *G6PD* | Chatham | 0 |
| chrX | 154533044 | rs137852339 | | *G6PD* | Kalyan-Kerala, Jamnaga, Rohini | 0 |
| chrX | 154533122 | rs137852327 | | *G6PD* | Ananindeua Hechi Viangchan, Jammu | 0 |
| chrX | 154533596 | rs137852318 | | *G6PD* | Bajo Maumere Seattle, Lodi, Modena, Ferrara II, Athens-like | 0 |
| chrX | 154533634 | rs137852346 | | *G6PD* | Aveiro | 0 |
| chrX | 154534102 | rs782757170 | | *G6PD* | Nanning | 0 |
| chrX | 154534125 | rs137852328 | | *G6PD* | A- 680T_376G | 0 |
| chrX | 154534157 | rs137852319 | | *G6PD* | Harilaou | 0 |
| chrX | 154534345 | rs137852326 | | *G6PD* | Cincinnati Minnesota, Marion, Gastonia, LeJeune | 0 |
| chrX | 154534348 | rs782754619 | | *G6PD* | Sibari | 0 |
| chrX | 154534387 | rs781865768 | | *G6PD* | Dagua | 0 |
| chrX | 154534389 | rs137852332 | | *G6PD* | Nilgiri Santiago | 0 |
| chrX | 154534390 | rs137852330 | | *G6PD* | Coimbra Shunde Vancouver | 0 |
| chrX | 154534419 | rs5030868 | | *G6PD* | Mediterranean, Dallas, Panama, Sassari, Cagliari, Birmingham | 0 |
| chrX | 154534438 | rs267606836 | | *G6PD* | Vancouver | 0 |
| chrX | 154534440 | rs5030872 | | *G6PD* | Malaga Santa Maria | 0 |
| chrX | 154534465 | rs137852343 | | *G6PD* | Nankang | 0 |
| chrX | 154534489 | rs137852331 | | *G6PD* | Taipei, Chinese-3 | 0 |
| chrX | 154534495 | rs137852314 | | *G6PD* | Mahidol | 0 |
| chrX | 154535176 | rs370918918 | | *G6PD* | Gond | 0 |
| chrX | 154535180 | rs782487723 | | *G6PD* | Shenzen | 0 |
| chrX | 154535187 | rs137852313 | | *G6PD* | Ilesha | 0 |
| chrX | 154535249 | rs782322505 | | *G6PD* | Cairo | 0 |
| chrX | 154535270 | rs78365220 | | *G6PD* | Crispim Salerno Pyrgos Vanua Lava | 0 |
| chrX | 154535277 | rs1050829 | | *G6PD* | 202G>A_376A>G_1264C>G A A- 202A_376G A- 680T_376G A- 968C_376G Acrokorinthos Ananindeua Mt Sinai Santa Maria Sierra Leone | 0 |
| chrX | 154535316 | rs5030870 | | *G6PD* | Sao Borja | 0 |
| chrX | 154535336 | rs267606835 | | *G6PD* | Vancouver | 0 |
| chrX | 154535342 | rs181277621 | | *G6PD* | Sierra Leone | 0 |
| chrX | 154535962 | rs782308266 | | *G6PD* | Lagosanto | 0 |
| chrX | 154535995 | rs782090947 | | *G6PD* | Murcia Oristano | 0 |
| chrX | 154535996 | rs137852349 | | *G6PD* | Namouru | 0 |
| chrX | 154536002 | rs1050828 | | *G6PD* | 202G>A_376A>G_1264C>G A- 202A_376G Asahi Hechi | 0 |
| chrX | 154536032 | rs137852315 | | *G6PD* | Metaponto | 0 |
| chrX | 154536168 | rs78478128 | | *G6PD* | Orissa | 0 |
| chrX | 154546061 | rs137852340 | | *G6PD* | Gaohe | 0 |
| chrX | 154546122 | chrX:154546122:C:A | | *G6PD* | Sinnai | 0 |
