# Supplementary material for: Development and validation of a pharmacogenomics reporting workflow based on the illumina global screening array chip
Source: Front Pharmacol. 2024 Mar 11;15:1349203. doi: 10.3389/fphar.2024.1349203 (PMC10961362; doi:10.3389/fphar.2024.1349203)
Supplement: Supplementary file 3 [file DataSheet1.PDF]

Atorvastatin

Atorvastatin, Atavor, Beatorva, Atorsan, Torvatec, Atswift, Atorvachol, Atorvon, Tulip, Atoris, Torvalip, Eturion, Lipitor, Actalipid, Removchol, Fastor, Stavinor, Genlipid, Atofit, Litorcom, Simtor, Tavora, Stator, Apo-Atorvastatin

START **MODERATE RECOMMENDATION**

GENE	GENOTYPE	PHENOTYPE
SLCO1B1	*15/*37	Possibly SLCO1B1 Decreased Function

Recommendation

Starting dose: <=40mg. Adjust based on guidelines. If >40mg needed, consider combination therapy.

Increased atorvastatin exposure as compared to normal and decreased function which may translate to increase myopathy risk.

Recommendations based on specific guidelines

**CPIC**	**Starting dose: <=40mg. Adjust based on guidelines. If >40mg needed, consider combination therapy. Prescriber should be aware of possible increase risk for myopathy especially for 40mg dose.**
DPWG	Choose alternative (rosuvastatin/pravastatin/fluvastatin). If not possible or no risk factors for myopathy: monitor muscle symptoms.

Caveat

Genetic variation is just one factor when prescribing statins. Rare variants may not be included in the genotype test. Patients with rare variants that reduce SLCO1B1 function may be incorrectly assigned a normal phenotype. Many patients' statin therapy is never restarted after Statin-related musculoskeletal symptoms. As a result, LDL cholesterol values are higher as is their risk for cardiovascular disease. The evidenced-based recommendations for genotype-guided statin therapy are focused on reducing the risk of SAMS.

Source

Publications related to local relevance for this drug-gene pair have not been published yet.

Decrease Starting Dose
Patient has altered metabolism rate or enhanced activation rate for drug indicated. Decreasing starting dose has shown to help prevent patient from experiencing adverse drug reaction.

Initiation
This drug-gene interaction recommendation is useful for initiation of this drug in naive patients. For patients who are already on stable dose or have used this drug before, effective drug monitoring and clinical judgement is more important.

This report was validated and generated automatically. No signature is required. Recommendations given in this report are made using a lab developed test which should not supersede clinical judgement or medical expertise.
