# Supplementary material for: Development and validation of a pharmacogenomics reporting workflow based on the illumina global screening array chip
Source: Front Pharmacol. 2024 Mar 11;15:1349203. doi: 10.3389/fphar.2024.1349203 (PMC10961362; doi:10.3389/fphar.2024.1349203)
Supplement: Supplementary file 4 [file Table5.DOCX]

**Supplementary Table 5: Inter-run concordance of genotype calls (SVs in *CYP2D6*).**

| **Sample** | ***CYP2D6* diplotype** | **Expected *CYP2D6***  **copy number** | **SV call** | | | **% concordance** |
| --- | --- | --- | --- | --- | --- | --- |
|  |  |  | **Replicate 1** | **Replicate 2** | **Replicate 3** |  |
| HG00276 | *4/*5 | 1 | Del | Del | Del | 100 |
| NA19226 | *2/*2XN | 3 | Dup | Dup | Dup | 100 |
| NA19109 | *2X/*29 | 3 | Dup | No-call | Dup | 100 |
| NA19207 | *2/*10/*XN | 3 | Dup | Dup | Dup | 100 |
| NA17244 | *2/*4/*XN | 4 | Dup | Dup | Dup | 100 |
| HG002 | *2/*4 | 2 | WT | No-call | WT | 100 |
