# Supplementary material for: Development and validation of a pharmacogenomics reporting workflow based on the illumina global screening array chip
Source: Front Pharmacol. 2024 Mar 11;15:1349203. doi: 10.3389/fphar.2024.1349203 (PMC10961362; doi:10.3389/fphar.2024.1349203)
Supplement: Supplementary file 6 [file Table4.DOCX]

**Supplementary Table 4: Concordance of PGx star alleles compared to pypgx (Lee et al. 2021).** Diplotype concordance for 4 genes with calls for 1KGP samples compared to pypgx. Percentages are reported with 95 % confidence intervals in brackets.

| **Gene** | **Size of reference call set** | **Diplotype callability (%)** | **Diplotype concordance (%)** |
| --- | --- | --- | --- |
| *DPYD* | 63 | 74.60  [62.66, 83.72] | 100.00  [92.44, 100.00] |
| *NUDT15* | 62 | 100.00  [ 94.17, 100.00] | 100.00  [94.17, 100.00] |
| *SLCO1B1* | 63 | 96.83  [89.14, 99.13] | 93.44  [84.32, 97.42] |
| *G6PD* | 63 | 100.00  [94.25, 100.00] | 100.00  [94.25, 100.00] |
